# Addressing osteoblast senescence: Molecular pathways and the frontier of anti‐ageing treatments

**DOI:** 10.1002/ctm2.70417

**Published:** 2025-07-18

**Authors:** Zhengdong Zhang, Pan Liu, Yu Song, Liang Ma, Yan Xu, Jie Lei, Bide Tong, Dingchao Zhu, Huaizhen Liang, Hongchuan Wang, Xingyu Zhou, Zixuan Ou, Junyu Wei, Hanpeng Xu, Di Wu, Shuchang Peng, Yifan Du, Zhi Du, Bingjin Wang, Zhiwei Liao, Wencan Ke, Kangcheng Zhao, Xiqin Xia, Lei Tan, Xiaobo Feng, Gang Liu, Shuai Li, Kun Wang, Cao Yang

**Affiliations:** ^1^ Department of Orthopaedics Union Hospital Tongji Medical College Huazhong University of Science and Technology Wuhan China; ^2^ School of Clinical Medicine Chengdu Medical College Chengdu China; ^3^ Department of Orthopaedics The First Affiliated Hospital of Chengdu Medical College Chengdu China; ^4^ Department of Endocrinology Hospital of Chengdu University of Traditional Chinese Medicine Chengdu China; ^5^ Department of Business English School of Foreign Languages Wuhan Business University Wuhan China

**Keywords:** ageing‐related signalling pathways, anti‐ageing strategies, bone microenvironment, osteoblast senescence, SASP

## Abstract

**Background:**

Osteoblast senescence is a central driverof age‐related osteoporosis. Accumulating evidence shows that counteractingthis senescence can substantially mitigate bone loss. In this review, we summarize the hallmarks of osteoblast senescence, the signaling pathways involved, and therapeutic strategies that target osteoblast senescence tocombat age‐related osteoporosis.

**Methods:**

Chronic diseases associated with ageingpose a significant threat to human health. Studies have shown that osteoporosisis closely linked to the ageing process of the body and the senescence ofosteoblasts within the bone microenvironment. Counteracting the senescence ofosteoblasts and maintaining the balance of differentiation, proliferation andfunction between osteoclasts and osteoblasts has been a key focus in the research of age‐related osteoporosis and bone loss. The biological behaviour andfunctionality of the osteoblast lineage related to senescence are modulated bya variety of targets, including signalling pathways, proteins and genes associated with ageing. This review aims to discuss the senescence‐related characteristics of the osteoblast lineage, dissect the interplay and mechanisms between it and ageing‐associated signalling pathways, proteinsand genes, as well as current strategies for the prevention and treatment ofosteoblast senescence.

**Conclusion:**

This review systematically examines the regulatory interactions among markers, therapeutic targets, and signalingpathways associated with osteoblast senescence, alongside current potential strategies for targeting this process. It provides more comprehensive information for future research into the complex mechanisms underlying age‐related osteoporosis driven by osteoblast senescence.

**Key points:**

- Osteoblast senescence is a key driver of age‐related osteoporosis, disrupting bone formation and homeostasis.
- Aging impacts osteoblasts through multiple pathways, including telomere shortening, genomic instability, SASP secretion, and others.
- Bone loss related to osteoblast senescence involves the activation and crosstalk of multiple signaling pathways.
- The options for combating and treating osteoblast senescence toachieve anti‐osteoporosis are numerous, but still challenging.

## Graphical abstract

Osteoblast senescence, a key driver of age‐related osteoporosis, disrupts bone formation and homeostasis through multiple pathways. Addressing this senescence to combat osteoporosis is challenging but holds promise.

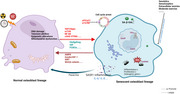

## 1 INTRODUCTION

In 1882, German biologist August Weismann introduced the ‘Wear and Tear’ theory. He posited that the human body and its cells are subject to damage through overuse and misuse, leading to cellular senescence. Weismann suggested that the life span of an organism is governed by the proliferative capacity of its somatic cells, a process shaped by genetic evolution. He noted that somatic cells across different species exhibit varying degrees of division potential, which ultimately dictates each species’ lifespan.^1^ In 1961, Leonard Hayflick and his colleagues made a seminal discovery regarding cellular senescence. They observed that when human fibroblasts were cultured in vitro, despite optimal growth conditions, the cells would eventually cease to divide after a certain number of divisions. This cessation ushered in an “irreversible” dormant state, a phenomenon now referred to as the Hayflick limit. They labelled this biological occurrence as cellular senescence.^2^

**FIGURE 1:**
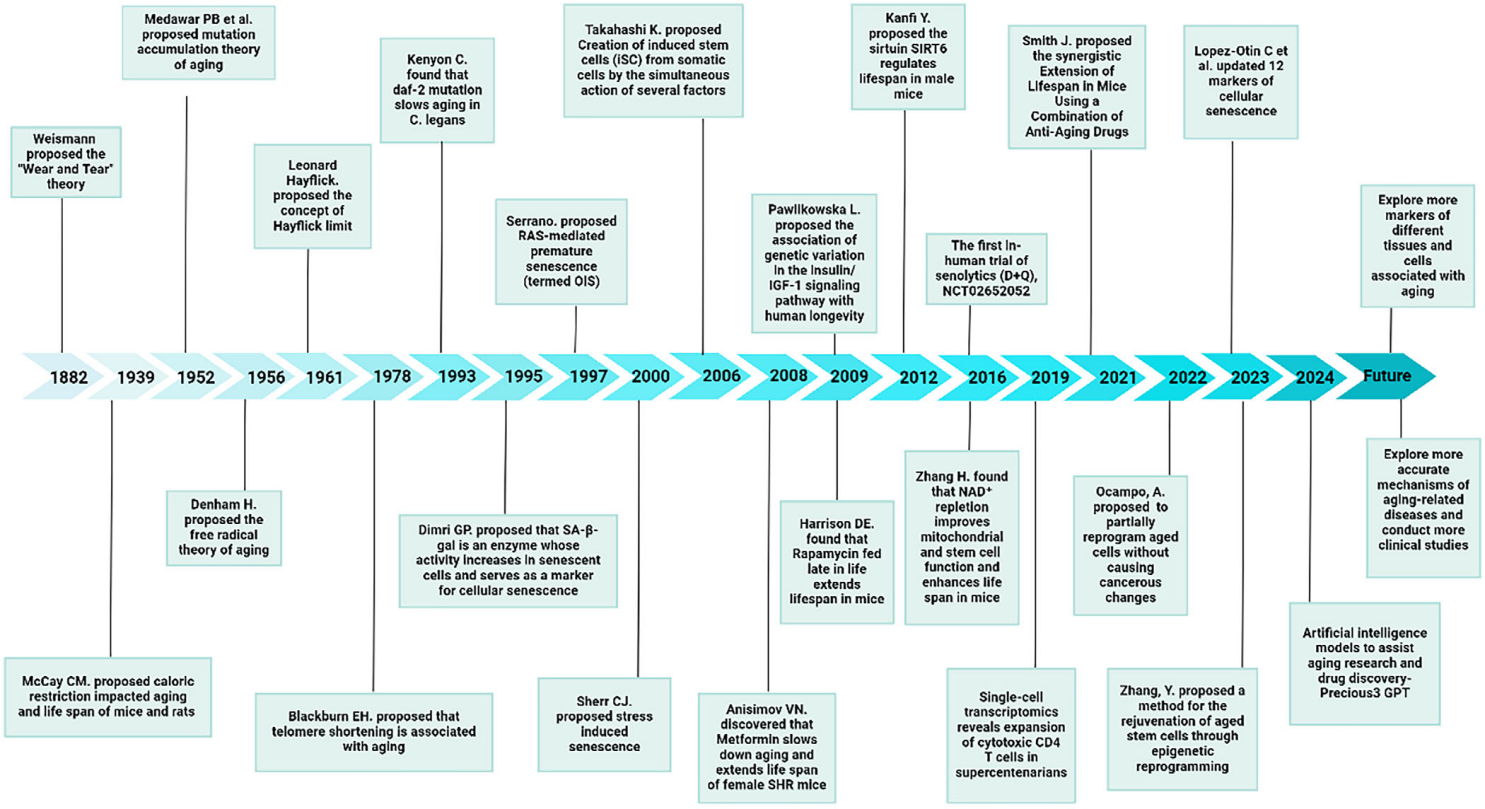
The timeline of important discoveries or outcomes in the field of ageing.

Over the past several decades, advancements in animal models, experimental techniques and equipment have profoundly broadened our comprehension of cellular and tissue senescence^3^, ^4^ (Figure 1). The homeostasis of bone tissue is maintained through a dynamic balance between the activities of osteoclasts and osteoblasts.^5^ With advancing age, osteoblasts exhibit diminished osteogenic capacity, characterised by reduced proliferative activity and impaired bone formation. Concurrently, bone resorption increases, disrupting the bone remodelling equilibrium and ultimately contributing to age‐related osteoporosis. In both developing and developed countries, ageing contributes to or coincides with diseases that increase economic and social burdens. Senile osteoporosis is often associated with a high risk of fractures, including spinal, hip and wrist fractures.^6^, ^7^ Reports indicate that the number of fractures (including hip, forearm and vertebral fractures) due to osteoporosis reaches approximately 9 million cases globally each year. It is estimated that by 2050, hip fractures in elderly men worldwide will increase by 310%, and those in elderly women will rise by 240%.^8^, ^9^

Current anti‐senile osteoporosis drugs have side effects such as teratogenicity, carcinogenicity, osteonecrosis of the jaw, increased hepatic and renal burden, cardiac side effects and efficacy rebound.^10^, ^11^, ^12^, ^13^ Therefore, it is imperative to deeply explore the pathophysiology and underlying mechanisms of senile osteoporosis to identify better and more stable drugs and methods for its prevention and treatment, and to clarify novel therapeutic approaches. This review covers the basic physiological and pathological mechanisms of cellular senescence and explores the potential causal relationship and mechanisms linking osteoblast senescence and osteoporosis. Our aim is to provide a theoretical foundation for the microscopic study of senile osteoporosis.

## 2 THE CHARACTERISTICS OF OSTEOBLASTS SENESCENCE

Cellular senescence refers to the gradual decline in cell proliferation and differentiation capabilities and physiological functions during the process of cells performing life activities. The phenomenon of cellular senescence was first observed in normal diploid cells that cease to proliferate after reaching the Hayflick limit.^2^, ^14^ This finding indicates that normal non‐malignant cells stop dividing in vitro after approximately 50 divisions.^15^ Its characteristics are manifested as the senescence‐associated secretory phenotype (SASP), prolonged cell cycle arrest, large molecule damage and metabolic disorder.^16^, ^17^

In 2013, Lopez‐Otín et al.^18^ summarised nine molecular, cellular and systemic markers of ageing: cellular senescence, genomic instability, telomere attrition, loss of proteostasis, epigenetic alterations, stem cell exhaustion, altered intercellular communication, mitochondrial dysfunction and deregulated nutrient‐sensing. With the continuous advancement of high‐throughput multi‐omics analysis techniques, the field of ageing research has gained further insights, and in 2023, three additional hallmarks of ageing were identified: disabled macroautophagy, chronic inflammation and dysbiosis.^19^

Before bone cells were recognised as essential for bone health, it was believed that all bone metabolism occurred on the bone's surface rather than within it.^20^, ^21^ The process of bone remodelling involves osteoclasts attaching to an old bone area, where they secrete proteases to digest the bone matrix and release minerals, forming Howship's lacunae. Subsequently, osteoblasts migrate to the site of the Howship's lacunae to secrete bone matrix, which then undergoes remineralisation to form new bone.^22^

Osteoblasts are derived from bone marrow mesenchymal stem cells (BMSCs) and pre‐osteoblasts and can further develop into bone lining cells and osteocytes, collectively referred to as osteoblast lineage cells.^23^ Osteoblast differentiation can be divided into three stages: proliferation, matrix maturation and mineralisation.^24^ Due to the short lifespan of osteoblasts (ranging from a few days to about 100 days), it is necessary to constantly replenish new cells to maintain the synthesis of the bone matrix. During this process, mesenchymal stem cells (MSCs) are believed to play a crucial role in bone regeneration. MSCs are involved in maintaining the balance between bone resorption and formation by controlling proliferation, differentiation and self‐renewal. Osteoblasts are often derived from the conserved programmatic step of MSCs to osteoblast progenitors to osteoblasts and are associated with the up‐regulation of runt‐related transcription factor 2 (RUNX2), activation of the Wnt pathway, and increased synthesis of bone morphogenetic proteins (BMPs).^25^ RUNX2 expression and nuclear translocation of β‐catenin are required in the early stages of osteogenic differentiation.^26^ Differentiation of pre‐osteoblasts into mature osteoblasts can be characterised by alkaline phosphatase (ALP) activity and expression. Osteoblasts exhibit different differentiation markers at various developmental stages, including ALP, type I collagen (Col1, in early bone progenitor cells), osteopontin (OPN, in immature osteoblasts) and osteocalcin (OCN, the Bglap2 gene, in mature osteoblasts). ALP represents an early marker of osteoblast differentiation, while OCN represents a late marker. At the end of bone formation, as the bone matrix matures, osteoblasts eventually follow three different paths: (i) becoming embedded in the bone matrix and differentiating into osteocytes, (ii) transforming into stationary, flat bone lining cells that cover the bone surface or (iii) undergoing apoptosis.^27^ Wu et al.^28^ used metabolomics analysis to find that the proliferation ability of osteoblasts, ALP activity and the levels of bone metabolism genes decreased with the increase in passage time. Here, we present several common cell sources of osteoblasts.

### 2.1 Mesenchymal stem cells

MSCs can be isolated from many tissues with varying osteogenic capabilities. Although both induced pluripotent stem cells (iPSCs) and embryonic stem cells (ESCs) can differentiate into osteoblasts,^29^ adult cells, predominantly MSCs, are readily available and thus become the commonly used seed cells for research.^30^ BMSCs are considered the prototypical MSCs. With the development and advancement of technology, other tissue sources, such as adipose‐derived MSCs (AdMSCs),^31^ and periodontal ligament stem cells (PDLSCs) have emerged. PDLSCs possess the potential to differentiate into osteoblasts.^32^ Other sources include human buccal fat pad‐derived mesenchymal stromal cells (hBFP‐MSCs), which can also be used as seed cells for bone formation. Recently, circulating osteogenic precursor (COP) cells have been identified as a relatively new entity in the field of stem cell and bone biology, with the potential for osteogenic differentiation both in vivo and in vitro. Due to their complex properties, the biology of COP cells is under investigation.^33^, ^34^ Previous studies have shown that the biological behaviour of rodent and human BMSCs changes with age including a loss of proliferation and differentiation potential, a loss of the ability to form bone in vivo and increased signs of ageing.^35^, ^36^

### 2.2 Mouse embryo osteoblast precursor cells

Mouse embryo osteoblast precursor (MC3T3‐E1) cells are derived from mouse cranial bone. Earlier studies showed that the proliferation rates of low‐passage cells (between Passage 20 and Passage 40) and high‐passage cells (between Passage 40 to Passage 60) were similar. However, the proliferation rate decreased in very high‐passage cells (between Passage 60 to Passage 95). The frequency of the S phase and the G2+M phase of the cell cycle in very high‐passage cells decreased and increased, respectively.^37^ These characteristics of MC3T3‐E1 cells make them ideal tools for studying the relationship between cellular senescence ageing and osteogenesis in vitro.

### 2.3 MG‐63 osteoblast‐like cells

MG‐63 cells are a human osteosarcoma‐derived cell line commonly used as a model for studying osteoblast‐like cells.^38^ Although they exhibit some characteristics of osteoblasts, they are not fully differentiated. However, with proper induction, MG‐63 cells can differentiate into more mature osteoblasts.

A recently proposed strategy, direct reprogramming (DR), bypasses the need for cells to transition through the stem cell phase. It converts patient somatic cells, often fibroblasts, directly into osteoblast‐like cells via DR.^39^ The feasibility of DR has been demonstrated in human foetal and neonatal foreskin fibroblasts.^40^ Although the current research is in its infancy, these different sources of fibroblasts transformed into osteoblasts by reprogramming hold important therapeutic prospects for diseases related to bone loss caused by ageing.^41^ The development of this new field may broaden and diversify the sources of osteoblast lineages.

## 3 MARKERS OF CELL SENESCENCE

Senescent cells undergo morphological changes, such as cell flattening, increased cell body volume and enlarged nuclear and nucleolar volumes.^42^, ^43^, ^44^ These morphological changes, while common, are not necessarily specific or characteristic features of cellular senescence due to the wide variation in normal cell morphology across different tissue sources. Microscopically, lysosomal expansion in senescent cells following mitosis has been observed both in vivo and in vitro.^45^, ^46^ Senescence‐associated β‐galactosidase (SA‐β‐Gal) is extensively employed as a biomarker for senescent cells.^47^, ^48^ The enzyme β‐galactosidase is predominantly found in lysosomes and is a highly abundant lysosomal enzyme, with an optimal pH of 4 in immortal or young cells. Upon cellular senescence, lysosomes expand and β‐galactosidase accumulates within them, becoming activated at pH 6. Consequently, the β‐galactosidase detected at pH 6 is referred to as SA‐β‐Gal. The expression of SA‐β‐Gal is commonly used to identify cellular senescence in both in vitro and in vivo tissues.^42^, ^49^, ^50^ However, under certain conditions, such as in vitro cell culture, SA‐β‐Gal staining may occur due to cell quiescence induced by serum starvation and contact inhibition.^51^, ^52^ It has also been reported that this enzyme's expression is intense in young nerve cells at pH 6,^53^ suggesting that SA‐β‐Gal is not the optimal marker for the specificity of cellular senescence. Another senescence‐related lysosomal enzyme, α‐fucosidase, is considered a more sensitive marker of senescence and can be used to detect senescence in cells with less sensitive SA‐β‐Gal expression.^54^, ^55^ Nevertheless, the detection of SA‐β‐Gal remains the most widely used method for cell senescence detection due to its simplicity, sensitivity and specificity. Recently, Wu et al.^56^ developed a tumour‐targeting fluorescence (FL)/photoacoustic (PA) probe using FL/PA imaging twin peaks, which has good sensitivity and spatial resolution. This probe is used for β‐galactosidase‐activated ageing tumour imaging, making the test results more accurate.^56^

For some time, senescent cells were thought to be closely associated with high expression of cell cycle regulators such as Cdkn2a (p16) and Cdkn1a (p21), which are involved in regulating cell cycle arrest. It was suggested that eliminating P16INK4A‐positive senescent cells could delay senescence‐related diseases.^57^ However, recent studies have shown that some senescent cells do not exhibit high p16 expression,^58^, ^59^ and conversely, not all cells with high p16 expression are necessarily senescent (for example, MSCs, macrophages and pancreatic β‐cells).^60^, ^61^, ^62^, ^63^ Moreover, lipofuscin, another hallmark of senescent cells, was isolated from 210 000‐year‐old human fossils.^64^ It is also considered a valuable marker of cell senescence. Angiopoietin‐like protein 2 (ANGPTL2) is a protein expressed in multiple organs of the human body.^65^ It plays a significant role in cellular senescence and has been identified as an ageing biomarker in various tissues, including the liver, skeletal muscle and skin.^66^, ^67^, ^68^

In addition, adipokines (resistin and leptin), dipeptidyl peptidase 4, oxidised vimentin, notch homolog proteins (NOTCH1 and NOTCH3), CD36 and intercellular adhesion molecule 1 have been proposed as biomarkers of biological ageing.^59^, ^69^ Nicotinamide adenine dinucleotide (NAD+) has antioxidant and anti‐ageing effects, and its levels decline during ageing.^70^ The decrease in NAD+ levels can also be regarded as a sign of cellular senescence.^71^ However, elevated NAD+ may have the opposite effect, promoting the SASP and senescence by interfering with AMP‐activated protein kinase (AMPK) and p53 activation and enhancing nuclear factor kappa B (NF‐κB) activity in senescent cells.^72^ This implies that NAD+ might assume distinct roles at various stages of ageing.

Because ageing‐related markers and other physiological and pathological features of the body and cells exhibit crossover or overlap,^73^ senescent cells of different tissue and cell origins are characterised by heterogeneity. As our understanding of ageing deepens, the age‐related heterogeneity of different tissues and cell sources is continually revealed, making the identification of ageing more precise and demanding. New technologies and methods are being developed to address this challenge by finding new and more specific biomarkers (such as single‐cell sequencing and EpiTrace sequencing through single‐cell chromatin accessibility)^74^, ^75^, ^76^ and optimising detection strategies. The future application of technologies such as artificial intelligence (AI), spatio‐temporal dynamics and cyclic markers is expected to increasingly clarify ageing markers across various tissues and cells.^59^

### 3.1 Telomere attrition

Telomere shortening is manifested as a spot‐like aggregation of chromatin structure, thereby inhibiting the expression of genes related to proliferation. This change is a consequence of impaired replication at the ends of deoxyribonucleic acid (DNA) sequences,^77^ a characteristic change known as senescence‐associated heterochromatin foci (SAHF).^78^ SAHF includes components such as retinoblastoma (RB), heterochromatin protein 1, trimethylated histone H3 Lys9, high mobility group protein A, the histone variant macroH2A (mH2A) and histone chaperones such as histone repressor A.^79^ Another chromatin feature of cellular senescence is senescence‐associated distension of satellites (SADS), which manifests as the decreased density of the centromeres and their surrounding heterochromatin.^80^, ^81^ SADS precedes the formation of SAHF and may be a potential and widespread marker of senescent cells.^58^, ^82^

Telomeres are unique structures situated at the tips of chromosomes, consisting of tandemly repeated DNA sequences and nuclear protein complexes bound to them.^83^ This structure effectively protects chromosome ends from exposure and fusion with adjacent chromosome ends.^84^ However, the telomeric DNA repeats shorten with each round of cell division.^85^ When telomeres shorten to a critical length, the stability of telomere DNA loops is compromised, leading to telomere ‘decapping’ and the formation of telomere dysfunction‐induced foci. This triggers the DNA‐damage response (DDR), causing cell cycle arrest and initiating the spontaneous senescence process.^86^, ^87^ Senescence‐related telomere shortening and ‘decapping’ are crucial factors in osteocyte senescence because they reduce cell proliferation.^88^ In a mouse model that exhibits accelerated ageing, impaired differentiation function of osteoblasts due to proliferation‐independent telomere dysfunction or defects in telomere maintenance molecules has been observed.^89^, ^90^, ^91^ For MSCs, telomere shortening limits their ability to differentiate into osteoblasts.^92^, ^93^

Telomere length in leukocyte is considered a predictor of ageing. Researchers have found that osteoporosis is associated with short telomeres in white blood cells in patients, while long telomeres in a female cohort are linked to higher bone density and a reduced risk of osteoporosis.^94^, ^95^, ^96^ However, another study did not support the notion that patients with osteoporosis experience accelerated telomere shortening and premature cell senescence.^97^ Therefore, studying the effect of age‐related telomere shortening on bone ageing requires further exploration of its underlying mechanisms and personalised characteristics. In general in vivo studies, the most commonly used animal model, the mouse, has longer telomeres and more widespread telomerase expression compared with humans.^87^ In summary, establishing a uniform standard for measuring telomere length between cells of different species and different cell types within the same species is challenging. Telomere shortening as a single indicator is difficult to use in clinical practice as a routine biomarker to predict ageing.^98^ Recently, Smoom et al.^99^ successfully created a ‘Telomouse’, a genetically engineered mouse model with shorter telomeres similar to human telomeres using clustered regularly interspaced short palindromic repeats‐CRISPR associated 9 (CRISPR–Cas9) gene editing technology, providing a new research tool for studying human telomere‐related diseases. With the development of gene editing technology, it is likely that more models similar to the ‘Telomouse’ will be developed in the future to better simulate the human ageing process.

### 3.2 Genomic instability

Genomic instability, where a cell's DNA fails to maintain its stability over time, is a recognised factor in ageing. The accumulation of genomic instability can result in the formation of DNA mutations and rearrangements, potentially leading to cell cycle arrest and the ageing process.^100^

Over the past few decades, it has been found that the level of DNA damage within cells and the ability to repair it are closely related to the average lifespan of a species, as well as the age at which primary cells separate from the individual.^101^, ^102^, ^103^ Exogenous (e.g., chemotherapeutic drugs, ultraviolet radiation, γ‐irradiation) and endogenous (e.g., oxidative damage, hyperproliferation, telomere attrition) stimuli activate the DDR, leading to irreparably damaged DNA and inducing cell senescence. At this point, cells can survive but not proliferate.^104^, ^105^, ^106^ Meanwhile, phosphorylated histone H2AX (γH2AX) lesions that signal DNA disruption accumulate at telomeres in human senescent fibroblasts.^107^, ^108^ γH2AX rapidly concentrates and anchors DNA repair proteins such as Nijmegen breakage syndrome gene (NBS1) and p53 binding protein 1 (53BP1) in the vicinity of telomere lesions.^81^ The emergence of γH2AX is linked to the activation of DNA damage effector kinases, such as checkpoint kinase 1 (CHK1) and checkpoint kinase 2 (CHK2).^109^, ^110^

P53, one of the key molecules in the cellular senescence pathway, is also activated by the DDR. When telomeres are damaged and the ends of chromosomes are exposed, cells recognise this as a double‐strand break, activating the p53 senescence pathway through DDR.^111^, ^112^, ^113^ This process involves the activation of ataxia telangiectasia mutated (ATM) and Rad3‐related protein, followed by the sequential activation of CHK2 and CHK1.^114^ These kinases phosphorylate multiple amino acid sites on the p53 protein, inhibiting its degradation and increasing its transcriptional activity, which further up‐regulates the expression of p21 and inhibits the cell cycle.^115^

Chemotherapeutic agents such as bleomycin, cisplatin or doxorubicin often cause persistent and irreparable nuclear DNA damage, resulting in DNA segments with chromatin mutagenesis (DNA‐SCARS).^116^ Several studies have explored the relationship between DNA damage and senescence in osteoblasts. Earlier research found a correlation between age‐related bone dynamics and decreased bone strength in male mice with defective DNA repair.^117^ Kim et al.^118^ discovered that osteoprogenitor cells isolated from elderly mice were arrested in the G1 phase and showed increased DNA damage and activation of p53. Approximately 70% of osteoprogenitor cells isolated from elderly mice showed γH2AX lesions, compared with 20% in young mice, and the SASP of senescent BMSCs and osteoblasts increased.^118^ Decreased levels of NAD+, radiation and genotoxic substances can cause DNA damage and cell senescence in osteoprogenitor cells, leading to loss of bone mass.^71^ Leucine (Leu) induces senescence in MC3T3‐E1 cells through DNA damage, negatively affecting the proliferation of osteoblasts.^119^ DNA damage in osteoprogenitor cells increases with age, suggesting a correlation between DNA damage in these cells and senescence.^120^ In turn, DNA damage‐induced senescence limits the number of bone progenitor cells and osteoblasts, leading to an increase in SASP and promoting osteoclast formation.^121^ Although there have been continuous advancements in understanding the relationship between DNA damage and cellular ageing, the specific mechanism by which DNA damage affects the complex process of osteoblast ageing still needs further elucidation.

### 3.3 Epigenetic alterations

Epigenetic alterations refer to changes in gene expression patterns that do not involve modifications to the underlying DNA sequence. These alterations play a significant role in the ageing process, as they can induce variations in gene expression that contribute to cellular and tissue dysfunction.^122^

A growing body of research indicates that epigenetic changes occur through three primary mechanisms: histone modification, DNA methylation and regulation by non‐coding microRNAs.^123^ Each of these mechanisms operates silently, causing alterations in the expression of genetic material without altering the underlying gene sequence.^124^ Therefore, changes in gene expression due to epigenetic alterations are believed to be responsible for the onset of premature ageing and the development of age‐related health risks.

Epigenetic alterations can induce various signs of ageing, cellular senescence, including loss of proteostasis, genomic instability, telomere shortening and mitochondrial dysfunction.^122^ The development of an epigenetic clock based on age‐related DNA methylation changes underscores the robust connection between epigenetic alterations and the ageing process.^123^ Growing evidence indicates that both senescent and cancer cells exhibit genomic and epigenomic changes driven by similar mechanisms.^125^ Studies have shown that epigenetic processes, such as DNA methylation, histone modifications and non‐coding RNA regulation, can influence the expression of genes associated with senescence, like p16 and p21, contributing to both osteoblast functional decline and the development of senescence phenotypes.^126^, ^127^ Understanding the mechanisms of epigenetic alterations and their impact on ageing provides a crucial pathway for developing strategies to mitigate age‐related diseases.

### 3.4 Loss of proteostasis

Protein homeostasis, or proteostasis, refers to a series of quality control mechanisms that maintain the balance of protein quality and quantity within the cell. This includes processes such as protein synthesis, folding, modification, transport, degradation and recycling.^128^ Proteostasis is essential for cell function because it ensures the proper function of proteins within the cell. At the same time, the stability of organelles is vital for maintaining cellular health and preventing disease.^129^, ^130^ As ageing progresses, the cellular mechanisms responsible for maintaining proteostasis gradually weaken. This decline results in the misfolding of proteins and the accrual of irreparable damage, which impairs the ability of chaperone proteins to correctly fold essential cellular proteins.^131^, ^132^ Over time, this cascade of events can culminate in cellular senescence and ultimately cell death.^133^

The peptide chains that constitute proteins are synthesised from mRNA through the action of ribosomes. The translation process is divided into three stages: initiation, elongation and termination. An abnormal translation process is the core cause of the loss of proteostasis.^134^ Stein et al. found that ageing can alter translation dynamics, leading to an imbalance in protein homeostasis due to the exacerbation of ribosomal stasis.^135^ This finding suggests that ageing may promote ribosome collisions, particularly in regions rich in polybasic amino acids, triggering the activation of ribosome‐associated quality control. Snieckute et al. found that reactive oxygen species (ROS) cause ribosome damage and activate the kinase ZAKα.^136^ This activation can trigger violent collisions of ribosomes within cells, contributing to conditions such as obesity and ageing.

The loss of proteostasis also affects the function and differentiation of osteoblasts. In osteoblasts, the loss of protein homeostasis leads to age‐related skeletal dysfunction.^137^ Studying the mechanisms underlying the imbalance of protein homeostasis may contribute to the development of innovative therapeutic strategies aimed at slowing down the ageing process and addressing diseases associated with ageing.

### 3.5 Deregulated nutrient‐sensing

Deregulated nutrient‐sensing is a key factor in ageing and disease. When the function of these sensing pathways is disrupted, a variety of metabolic diseases may arise. Cells have evolved complex mechanisms to sense and respond to the availability of nutrients such as glucose, amino acids and lipids. Key nutrient‐sensing pathways include the mechanistic target of rapamycin (mTOR) pathway, the insulin/insulin‐like growth factor (IGF‐1) signalling pathway and AMPK, all of which regulate metabolism and influence the ageing process.^138^, ^139^, ^140^ High‐calorie diets or diets rich in certain nutrients can chronically activate nutrient‐sensing pathways, potentially leading to metabolic disorders and accelerated ageing.^141^ For example, high‐fat diets (HFD) can activate mTOR.^142^ In contrast, nutrient limitation or diets low in protein can extend healthspan by activating the AMPK–silent information regulator family protein (SIRT) pathway.^143^

During the ageing process, nutrient‐sensing pathways may fail to respond appropriately to changes in nutrition, leading to an imbalance in cellular metabolism and a reduction in the efficiency of energy production.^144^ Autophagy, the cellular process by which damaged and aged organelles and misfolded proteins are removed, is influenced by nutrient‐sensing.^145^ If autophagy is impaired, it may lead to the accumulation of damaging cellular components, which are associated with ageing and various degenerative diseases.^146^

Deregulated nutrient sensing can also trigger chronic low‐grade inflammation, a condition linked to metabolic disorders such as type 2 diabetes mellitus and obesity.^147^ Long‐term exposure to high glucose levels in the body leads to pathological states of insulin resistance, diabetes and bone loss.^148^ In vitro studies have shown that high glucose (22–30.5 mM) results in increased production of ROS and decreased proliferation and mineralisation in the osteoblast lineage.^149^, ^150^

HFD rich in saturated fatty acids, along with high‐calorie diets, can lead to increased inflammation and oxidative stress, which in turn affect bone density and increase the risk of fractures.^151^, ^152^ However, Minematsu et al.^153^ reported that 12‐month‐old Wistar male rats fed a high‐fat/high‐sucrose diet for 6 months showed an increase in trabecular and cortical bone mass, though no in vitro experiments were conducted in this study. Sex dimorphism is also an important factor that influences the regulation of nutrient sensing in bone metabolism during ageing.^154^ These findings highlight the complexity of nutrient sensing and its impact on bone metabolism during ageing.

### 3.6 Mitochondrial dysfunction

Mitochondria are essential cellular organelles that promote basic energy conversion within the cell and are responsible for the production of the energy required for cellular functions through the oxidative phosphorylation (OXPHOS) process.^155^, ^156^ They are crucial for many cellular processes, including adenosine triphosphate (ATP) production, β‐oxidation of fatty acids, apoptosis and cell senescence.^157^ The main characteristics of mitochondrial dysfunction include changes in mitochondrial DNA (mtDNA), defects in mitochondrial biogenesis and dynamics, disruptions in mitochondrial autophagy, impairments in OXPHOS and excessive accumulation of ROS.^158^, ^159^ Furthermore, mitochondrial dysfunction can also alter intercellular crosstalk by influencing various signalling mechanisms, such as the release of mitochondria‐derived vesicles (MDVs) and metabolite signalling.^160^ Mitochondrial dysfunction‐associated senescence is typified by a reduction in the NAD+/nicotinamide adenine dinucleotide ratio and induces cell cycle arrest through AMPK‐mediated activation of p53.^161^

Current studies have confirmed that mtDNA point mutations and deletions accumulate with age.^162^ In mammals, an increase in mtDNA mutations in tissues like the brain, skeletal muscle and heart is observed with ageing, correlating with the severity of declines associated with ageing.^163^, ^164^ Mitochondrial quality control (MQC) is essential for regulating ROS production and levels within cells.^165^ The primary role of MQC is to maintain cellular homeostasis by coordinating processes such as mitochondrial fission, fusion, mitophagy and mitochondria‐induced cell death, thereby delaying the ageing process.^166^

In osteoblasts, the dynamic changes in mitochondria are crucial to the process of bone formation. Suh et al. found that mitochondria release MDVs during osteogenesis in osteoblasts.^167^ These MDVs can be internalised by surrounding osteoblast precursor cells through a specific cellular uptake mechanism, promoting their differentiation. Furthermore, the CD38/ cyclic ADP‐ribose signalling pathway enhances mitochondrial fission and the formation of specific mitochondrial structures, such as ring‐shaped mitochondria (donuts). Additionally, the regulation of mitochondrial division by the optic atrophy 1 (OPA1) protein contributes to increased bone formation.^167^

Mitochondrial dysfunction can impede the differentiation of osteoblasts from progenitor cells to mature forms and disrupt the signalling mechanisms between osteoblasts and osteoclasts.^168^, ^169^ This disruption can ultimately throw off the balance of bone metabolism, potentially leading to osteoporosis.^170^ The inner mitochondrial membrane contains the ATP synthase complex V and the respiratory chain complexes I, II, III, IV, all of which are crucial for OXPHOS.^171^ Enhancing OXPHOS in osteoblasts may augment bone anabolic effects.^172^

Complex I and complex III are acknowledged as the primary sources of ROS generation in the mitochondrial electron transport chain.^173^, ^174^ At low concentrations, ROS serve a pivotal role as signalling molecules, influencing key cellular processes such as differentiation, apoptosis and proliferation.^175^ However, when ROS accumulate in excess, they are linked to oxidative stress, which is detrimental to osteoblasts and osteocytes, potentially inducing apoptosis and impairing bone mineralisation and osteogenesis.^176^ Moreover, the overproduction of ROS can intensify these negative effects by stimulating the differentiation of osteoclasts and macrophages, consequently hastening bone loss.^177^ These findings underscore the importance of mitochondrial function in bone health. They imply that interventions aimed at bolstering mitochondrial performance could be beneficial in preserving osteoblast vitality and enhancing bone remodelling, particularly in the context of ageing‐related bone disorders.

### 3.7 Ageing caused by oncogenes

Cell cycle arrest triggered by oncogene activation is regarded as a pivotal mechanism for curbing tumour progression. This process can initiate a form of cellular senescence known as oncogene‐induced senescence (OIS), which acts as a tumour suppressor response to impede the proliferation of cells with the potential to become cancerous.^178^

The pathways involving p53/p21 and P16 are predominantly responsible for mediating cellular senescence, and their activation is widely recognised as a molecular signature of this state.^179^ Among the numerous downstream effectors of p53, the protein p21 is particularly instrumental in driving cell senescence.^180^, ^181^ P21 contributes to G2/M cell cycle arrest by impeding the activity of cyclin‐dependent kinase (CDK) 1. Additionally, it suppresses the activity of CDK2 and CDK4, leading to a decrease in the phosphorylation of the RB protein (pRb). This inhibition prevents cells from transitioning into the S phase of the cell cycle, thereby maintaining the senescence state.^182^, ^183^

OIS is typically orchestrated by the tumour suppressors p16 and alternate reading frame, which are encoded at the CDK inhibitor 2A (CDKN2A) locus. Their action is instrumental in enforcing cell cycle termination.^184^, ^185^ P16, acting as an inhibitor of the CDKs CDK6 and CDK4, phosphorylates and inactivates the RB1 protein, thereby playing a crucial role in modulating the ageing process.^186^

Recent investigations have elucidated that the protein cold shock domain containing E1 (CSDE1) fosters OIS via two distinct molecular pathways. First, it augments the stability of mRNA transcripts encoding SASP factors, thereby amplifying their expression. Second, it exerts an inhibitory effect on the translation of Y‐box binding protein 1 (Ybx1) mRNA, a process integral to the regulation of OIS.^187^

### 3.8 Stem cell exhaustion

The ageing process is characterised by a gradual yet profound deterioration in the regenerative potential of tissues, a phenomenon intimately connected to the concept of stem cell exhaustion.^188^ Stem cell exhaustion is manifested by a continuous reduction in both the abundance and functionality of stem cells. These cells serve as the cornerstone of tissue maintenance and repair, playing a crucial role throughout an organism's lifespan.^189^, ^190^ This decline in stem cell vigour is an inherent aspect of ageing, affected by diverse factors that can compromise the cells' structural integrity and operational efficiency.

One of the principal drivers of stem cell exhaustion is the erosion of telomeres—the protective caps at the chromosome termini. With each round of cell division, telomeres shorten incrementally, culminating in a critical length that precipitates cell cycle arrest and senescence.^191^ This phenomenon is especially deleterious for stem cells, given their reliance on a robust capacity for division to replenish tissue reserves. The rate at which telomeres shorten can be intensified by oxidative stress and various environmental stressors, hastening the progression of stem cell exhaustion.^192^, ^193^ Excessive ROS can inflict damage upon stem cells, leading to mutations, compromised functionality and a diminished capacity for self‐renewal.^194^ Over time, chronic exposure to ROS accelerates the senescence process and the exhaustion of stem cell populations.^195^ DNA damage is an unavoidable byproduct of cellular metabolism, with the impact potentially magnified by exposure to environmental mutagens.^196^ Although young stem cells are equipped with robust DNA repair mechanisms, these can diminish in efficacy with advancing age.

The waning regenerative capacity of tissues, attributable to stem cell exhaustion, concurrently heightens the vulnerability to a spectrum of age‐related maladies. As the reservoir of stem cells necessary for repairing tissue damage and replenishing lost cells diminishes, tissues become increasingly susceptible to the ravages of chronic inflammation, metabolic dysregulation and a host of other pathological cascades.^197^, ^198^ The molecular and cellular mechanisms underlying stem cell exhaustion are complex and multifaceted. They involve changes in the epigenetic landscape, alterations in cellular metabolism and a decline in the production of growth factors and cytokines that support stem cell function.

The interaction between BMSCs and osteoblasts is bidirectional. Senescent osteoblasts can release factors such as the SASP that impair the function of MSCs, leading to stem cell failure.^199^ Correspondingly, a depleted MSCs may have a decreased ability to differentiate into osteoblasts, thereby exacerbating the impediments to bone formation.^200^, ^201^ This vicious cycle can lead to an imbalance in bone remodelling, aggravating bone resorption rather than bone formation.

### 3.9 Senescence‐associated secretory phenotype

Most cells that enter senescence exhibit the production of the SASP. The SASP is not a static entity; it evolves over time.^202^ Despite having ceased to divide, senescent cells retain metabolic activity and are capable of secreting SASP components.^203^ The SASP is one of the key markers of senescent cells and has local paracrine and systemic effects at a distance, mediating the pathological and physiological impacts of many senescent cells.^57^, ^204^ The constituents of the SASP are diverse, primarily encompassing growth factors, chemokines, proinflammatory cytokines and extracellular matrix (ECM) remodelling enzymes, collectively referred to as the SASP or senescence messaging secretome.^205^ Key proteins associated with the SASP include tumour necrosis factor (TNF)‐α, monocyte chemoattractant proteins (MCP)‐1 and ‐2, interleukins (IL) such as IL‐6, IL‐1α, IL‐7 and IL‐8, plasminogen activator inhibitor, growth‐regulated oncogenes (GRO)‐α, GROβ and GROγ, IGF‐7, macrophage inflammatory protein‐1α and matrix metalloproteinases (MMP) such as MMP‐1, MMP‐10 and MMP‐3 and so on.^206^, ^207^ Additional components of the SASP include exosomes, vesicles, specific DNA fragments, a various of microRNAs and other non‐coding RNAs.^208^ These factors contribute to the complex and dynamic nature of the SASP, influencing cellular crosstalk and modulating the tumour microenvironment, tissue repair and the ageing process.

Subsequent research has largely reached a consensus: the unchecked accumulation of senescent cells, if not effectively removed by the immune system, can result in the persistent secretion of the SASP, potentially contributing to a variety of age‐related diseases.^58^ Initially, SASP accumulates within the body and its organs, altering the tissue microenvironment. This shift can precipitate chronic inflammatory responses, both directly and indirectly, accelerating the senescence process of neighbouring normal cells.^209^

It is recognised that the secretion of the SASP can be subject to both inhibition and promotion mechanisms.^210^, ^211^ Moreover, the regulation of SASP may also be associated with oxidative stress and DNA damage.^212^ It is noteworthy that certain SASP components, including colony‐stimulating factor 1, IL‐8 and C‐C motif chemokine ligand 2 (CCL2), possess the capacity to recruit immune cells. This recruitment facilitates the immune system's ability to target and potentially eliminate senescent cells, thereby contributing to a self‐regulated clearance process.^213^

Farr et al.^214^ discovered that during the onset of osteoporosis, the secretion of SASP components, such as IL‐6, MCP‐1 and IL‐8, by senescent cells within the bone microenvironment, adversely affects the differentiation capacity of pre‐osteoblasts. This impairment results in a cascade of effects, including reduced osteoblast formation, diminished bone mineralisation and an increase in osteoclast formation.^214^ Xu et al.^215^ induced senescence in MLO‐Y4 cells (murine long‐bone osteocyte) using a 2 Gy γ‐ray irradiation and observed an up‐regulation in the expression of 72 SASP factors. They further noted that senescent osteocytes, through paracrine signalling, down‐regulated the expression of the RUNX2 in BMSCs.^215^ Senescent cells in bone tissue can interfere with neighbouring cells and release SASP factors locally and systemically.^216^ It is possible that senescent osteocytes and their secreted SASPs are significant contributors to the degenerative differentiation of BMSCs.

Although multiple pathways have been reported to initiate and regulate senescent cells, the exact mechanisms that govern the SASP remain not fully understood. In certain specific environments and at particular times, SASP can have beneficial effects on the organism.^217^, ^218^ This is attributed to the considerable heterogeneity and the high degree of cell‐specific, time‐specific and stress‐specific properties associated with the SASP.

### 3.10 Disabled macroautophagy

In the 1960s, the concept of ‘autophagy’ was first articulated to describe the cellular process of self‐digestion.^219^ A significant leap in our understanding of this phenomenon occurred in the 1990s with the discovery of autophagy‐related protein (ATG) genes in the yeast species *Saccharomyces cerevisiae*.^220^ Autophagy encompasses three principal modalities: macroautophagy, microautophagy and chaperone‐mediated autophagy.^221^

In recent years, the impairment of macroautophagy has come to be recognised as a hallmark of ageing, intricately connected with cellular senescence. Macroautophagy is a vital cellular mechanism that facilitates the degradation and recycling of cellular constituents, thereby ensuring the maintenance of cellular homeostasis and function.^222^ Impaired macroautophagy has been pinpointed as a key factor in the onset of cellular senescence, resulting in the accumulation of damaged cellular elements and the subsequent triggering of senescence‐associated pathways.^19^, ^223^

Autophagy plays a pivotal role in maintaining the characteristics and differentiation potential of stem cells, attributes that may decline as these cells undergo senescence.^224^ In MSCs and osteoblasts, a decrease in autophagy with age has been observed. Enhancing autophagy not only mitigates senescence effects in MSCs but also enhances their osteogenic differentiation and proliferation capabilities.^225^ During the early phases of osteogenic differentiation, MSCs demonstrate a transient decrease in the autophagosome marker, microtubule‐associated protein light chain 3‐II (LC3‐II) within the first 12 h. This reduction suggests that the formation of autophagy vacuoles might provide essential energy substrates required for the differentiation process.^226^ In contrast, the impairment of autophagy in osteoblasts can result in diminished mineralisation, reduced bone mass and the premature ageing of bone tissue.^227^ Furthermore, the mineralisation process in osteoblasts is contingent upon the involvement of ATGs such as beclin 1 (BECN1), ATG5 and ATG7.^228^ These proteins are integral to the autophagic flux that contributes to bone formation and the maintenance of bone health.

The impairment of macroautophagy has also been associated with additional hallmarks of ageing, including chronic inflammation and microbial dysbiosis, underscoring its pivotal role in the ageing process. The established connection between compromised macroautophagy and cellular senescence highlights the significance of preserving adequate autophagic activity to prevent cellular senescence and associated dysfunction.

### 3.11 Chronic inflammation

Prior research has established that chronic, low‐grade inflammation can precipitate premature ageing in mice, leading to a decline in the regenerative capacity of key organs.^229^ The elimination of senescent cells from tissues has been demonstrated to reduce the expression of pro‐inflammatory cytokines, thereby extending the lifespan of mice, whether they age naturally or at an accelerated pace.^230^ Similarly, interventions such as blocking TNF‐α, inhibiting the nucleotide‐binding oligomerisation domain‐like receptor protein 3 (NLRP3), or genetically deleting apoptosis signal‐regulating kinase 1 have been demonstrated to attenuate inflammation and improve age‐related diseases in aged mice.^231^

Inflammatory mediators can directly or indirectly suppress osteoblast activity and impede bone formation.^232^ The NF‐κB signalling pathway, when engaged in inflammatory responses, can drive an senescence phenotype in skeletal stem/progenitor cells.^233^ Age‐related inflammation, orchestrated by NF‐κB and Toll‐like receptor signalling, diminishes the quantity and functionality of bone progenitor cells, adversely impacting fracture repair processes.^199^ Moreover, in diabetic patients, persistent high glucose levels continuously fuel the generation of ROS, inducing a state of low‐grade systemic inflammation. This condition hinders the proliferation, differentiation and mineralisation of osteoblasts, culminating in diminished bone formation.^148^

There exists a robust correlation between chronic inflammation and cellular senescence. As ageing advances, the accumulation of senescent cells across various tissues sets up a positive feedback loop with inflammation. This interplay results in chronic aseptic inflammation, which, in turn, accelerates the ageing process.^234^

Molecular and cellular therapies that modulate the behaviour of inflammatory cells and bone stem cells are emerging as promising therapeutic targets. These interventions aim to enhance osteogenic differentiation or improve osteoblast function, thereby presenting a novel approach to treating bone diseases associated with ageing and inflammation.

### 3.12 Dysbiosis

The interplay between cellular senescence and dysbiosis, particularly within the gut microbiome, has become a significant focus in the study of ageing and its associated diseases. Dysbiosis, characterised by an imbalance in the gut flora, has been found to be positively correlated with cellular senescence and heightened inflammation. Research indicates that enteric dysbiosis can suppress osteoblast activity and stimulate osteoclast function, resulting in disruptions to bone homeostasis.^235^ The gut microbiota–gut–bone axis has been proposed as a potential mechanism linking dysbiosis to bone health and the onset of cellular senescence.^152^, ^236^ Sharma et al.^237^ have illuminated the promising capacity of probiotic bacteria to modulate cellular senescence. They have put forth the notion that investigating the combined effects of probiotics with plant polyphenols may yield innovative probiotic‐based strategies aimed at combating cellular senescence.^237^

Numerous studies have delved into the complex relationship between dysbiosis and cellular senescence, particularly concerning osteoblast function and skeletal health. Queiroz and colleagues investigated the role of the angiotensin‐converting enzyme 2/angiotensin 1–7/mas receptor axis in osteoblasts and osteoclasts. Their research focused on alveolar bone remodelling induced by dysbiosis in rats,^238^ underscoring the significance of unravelling the molecular mechanisms behind dysbiosis‐induced bone resorption and remodelling processes. In another study, Behera et al.^239^ examined the impact of probiotic supplementation on bone formation in obese mice, revealing that it stimulated bone formation through histone methylation. This research highlights the therapeutic potential of probiotics in addressing bone disorders associated with dysbiosis. Chen et al.^240^ discovered that a HFD can lead to the premature senescence of BMSCs, reducing their proliferative capacity and bone‐forming ability. The study also found that administering vitamin D receptor (VDR) activators to HFD‐fed mice could reverse BMSCs senescence, decrease intracellular ROS levels, maintain mitochondrial function and bolster bone formation.^240^

Studies have indicated that dysbiosis impacts the biological characteristics of stem cells and osteoblasts, emphasising the importance of understanding and addressing microbiome imbalances, particularly in the context of ageing. Future research may provide further insights into the mechanisms through which dysbiosis influences osteoblast functionality.

### 3.13 Altered intercellular communication

Intercellular communication is essential for the orchestration of cellular activities within tissues and is paramount in sustaining homeostasis. This communication is facilitated through diverse mechanisms, including the secretion of signalling molecules such as cytokines and growth factors, direct cell–cell contact, changes in the microenvironment and the transmission of information via extracellular vesicles (EVs) like exosomes and microvesicles.^241^, ^242^ However, as age advances, the efficacy and precision of these communication pathways may diminish, precipitating a cascade of events that can exacerbate the ageing phenotype.^243^

In the context of osteoblast senescence, altered intercellular communication, plays a significant role. As osteoblasts age, their ability to communicate effectively with surrounding cells, including osteoclasts and osteocytes, becomes compromised. This disruption in communication can lead to imbalances in bone remodelling, contributing to conditions such as osteoporosis.

### 3.14 Changes in bone marrow adipose tissue

The balance between adipose and osteoblast cells is a primary concern in bone metabolism. In mammals, bone marrow is a unique organ where bone tissue and adipose tissue coexist, and it is the only tissue where adipocytes and osteocytes are in close proximity. The presence of bone marrow adipose tissue (BMAT) within the bone makes it more challenging to study than adipose tissue located outside the bone. This characteristic has resulted in a scarcity of research data, which in turn stimulates ongoing development in new technologies and research methodologies. Pierre et al. originally studied fat from iliac bone biopsies, investigating its use to replace bone marrow components in adult patients with osteoporosis.^226^ Meunier et al. speculated that osteoporosis itself causes bone marrow to be replaced by fat.^244^ This theory has been repeatedly confirmed through dual‐energy computed tomography, bone biopsies and magnetic resonance imaging in vivo.^245^, ^246^

Distinct from brown, white and beige adipocytes, marrow adipocytes have been identified as a separate category within the adipocyte lineage.^247^ Decades of research have shown that an increase in BMAT is associated with a decrease in MSCs during ageing and disease. Since MSCs are the common precursor cells for both osteoblasts and adipocytes,^248^ the dynamic balance between these cell types is crucial for maintaining bone health. BMAT is dynamic and can be influenced by environmental, nutritional and hormonal stimuli.^249^ In the ageing microenvironment, the proportion of MSCs differentiating into adipocytes increases, and BMAT depletes the limited pool of MSC.^250^ The equilibrium between MSCs differentiating into adipocytes or osteoblasts is regulated by key transcription factors such as peroxisome proliferator‐activated receptor γ (PPARγ), RUNX2 and CCAAT/enhancer binding protein α (C/EBPα).^251^, ^252^ However, the secretory factors associated with adipocytes that potentially regulate bone resorption or bone formation remain largely unknown.

Before a certain age, BMAT does not appear to have obvious side effects on bone structure. However, as the body ages, the content of BMAT seems to be inversely proportional to the amount of bone mass.^253^ Other studies have presented the opposite view, suggesting that high bone mass and high BMAT can coexist,^254^ and that an increase in BMAT does not lead to age‐related bone loss.^255^ This controversy introduces new challenges to research, indicating that the interplay between BMAT metabolism and bone mass during the ageing process requires further investigation.

Research has indicated that BMAT is linked to DNA damage; alleviating oxidative stress‐induced DNA damage can mitigate BMAT and enhance bone structure following radiation exposure.^256^ In vivo studies have shown that BMAT is reduced in aged and irradiated bones following the removal of senescent cells.^257^ Factors originating from adipocytes in BMAT can hinder the transformation of BMSCs into osteoblasts and influence bone remodeling.^258^ Meanwhile, the increased expression of PPARγ encourages BMSCs to differentiate into adipocytes while suppressing osteoblast differentiation.^259^ Bone marrow adipocytes from postmenopausal women can produce palmitic acid, which is lipotoxic to osteoblasts, leading to functional inhibition, defective autophagy and increased apoptosis of osteoblasts.^260^ Wnt signalling and Hedgehog signalling inhibit the differentiation of BMSCs into adipocytes and promote their differentiation into osteoblasts. However, these signalling pathways generally decrease with ageing, leading to adipose tissue accumulation in the bone marrow cavity and threatening the survival of osteoblasts.^261^, ^262^ Furthermore, bone marrow adipose lineage cells‐derived receptor activator of NF‐κB ligand (RANKL) regulates osteoclast formation.^263^

**FIGURE 2:**
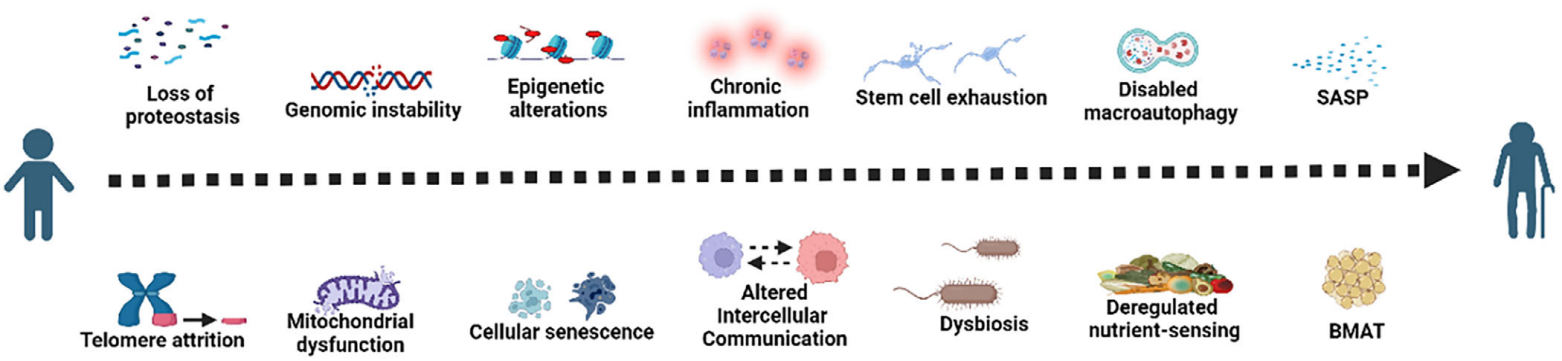
The main hallmarks of osteoblast senescence.

On the other hand, ageing promotes lipid redistribution in the body and induces a persistent chronic low‐grade inflammatory state.^264^ The ‘bystander effect’ of this continued proinflammatory status may negatively affect the proliferation and differentiation abilities of surrounding cells, significantly impairing the osteogenic differentiation ability of non‐senescent BMSCs through the SASP.^265^ Musashi2 (Msi2) can promote the differentiation of BMSCs into osteoblasts and inhibit their differentiation into adipocytes. The decreased expression of Msi2 in BMSCs of aged mice suggests that the reduced expression of Msi2 during ageing shifts the balance of osteogenesis/adipogenesis towards adipogenesis, leading to osteoporosis.^266^ A recent study found that cellular senescence is an important early trigger for the differentiation of BMSCs into adipocytes during bone ageing. RNA‐seq data showed that BMAT‐related genes were up‐regulated in C57BL/6 mice 3 weeks after radiation exposure. Inhibition of the SASP can regulate BMAT, while targeting individual adipokines can also direct the fate of BMSCs towards osteogenic differentiation.^267^ Given this, we boldly listed BMAT as a marker of senescence in osteoblast cell lines, aiming to provide a reference for future research in the fields of ageing and bone metabolism (Figure 2).

Although the detailed mechanism between BMAT and osteogenesis is not fully understood, these results suggest that the inhibition of lipogenic differentiation of BMSCs may be one of the important strategies for treating senile osteoporosis.

## 4 SIGNALLING PATHWAYS ASSOCIATED WITH OSTEOBLAST SENESCENCE

**FIGURE 3:**
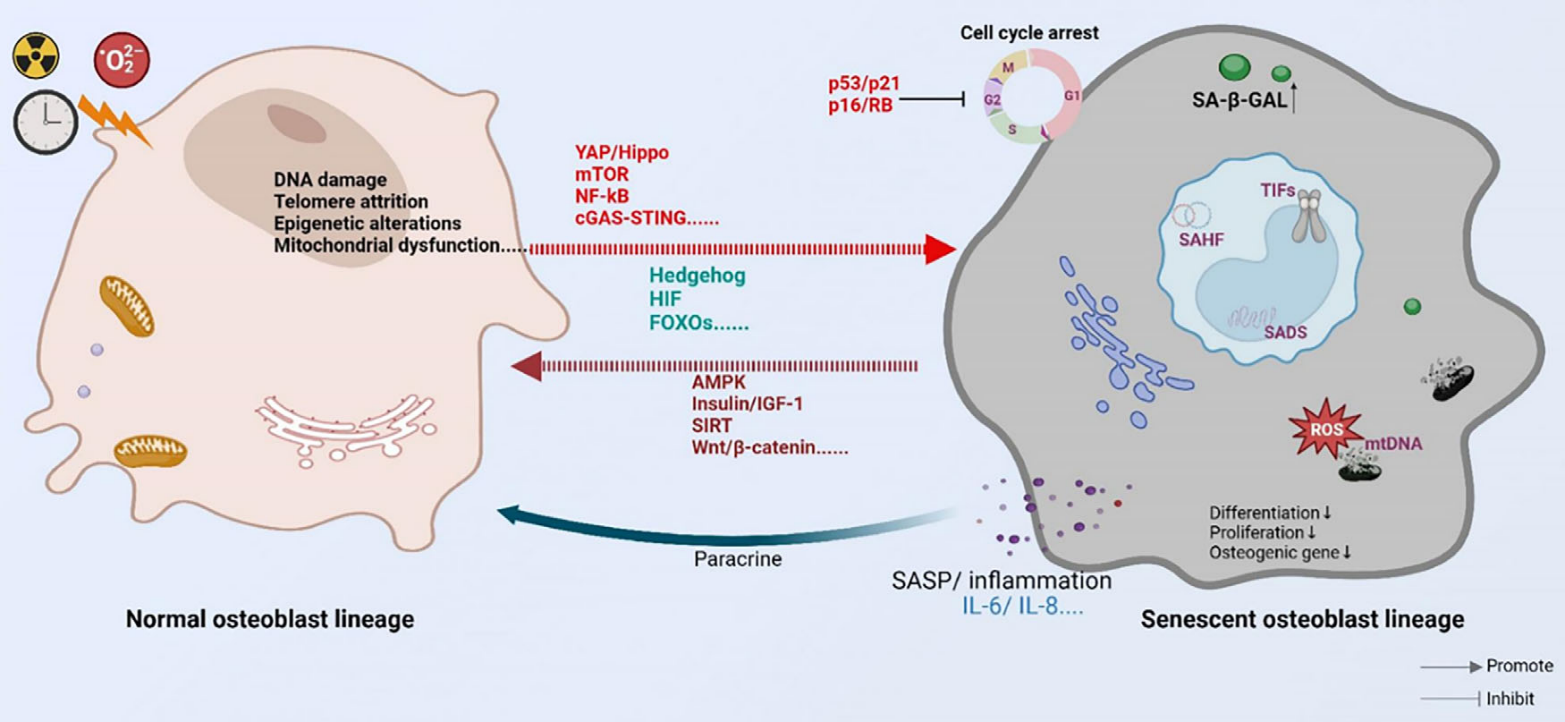
The diagram illustrates the process of osteoblast senescence.

The differentiation, proliferation, maturation and mineralisation of osteoblast lineages depend on the coordinated action of multiple signalling pathways. Ageing affects not only the ability of osteoblasts to proliferate and differentiate into osteogenic cells but also impacts the homeostasis of the skeletal system (Figure 3). Although studies on the effects of ageing on osteoblast lineages are not yet comprehensive, several signalling pathways have been identified that play a role in age‐related changes to osteoblast lineages.

### 4.1 mTOR signalling pathway

The mTOR protein, an evolutionarily conserved Ser/Thr kinase, functions as the catalytic subunit in two separate complexes: mTOR complex 1 (mTORC1) and mTOR complex 2 (mTORC2).^268^ mTORC1 is primarily believed to regulate cell growth, senescence, proliferation, apoptosis, energy metabolism and autophagy, while mTORC2 mainly controls cell survival and cytoskeleton reorganisation.^269^

Gharibi et al.^270^ reported that BMSCs cultured with inhibitors of AKT or mTOR could stably maintain their osteogenic potential and anti‐senescence phenotype expression. Additionally, rapamycin treatment of BMSCs showed higher osteogenic differentiation ability and inhibited senescence by reducing ROS and p53 expression levels via autophagy activation.^271^, ^272^ Liu et al.^273^ found that the expression of Leu‐rich repeat containing 17 (LRRc17) was significantly increased in senescent BMSCs. Knockdown of LRRc17 activated mitochondrial autophagy by inhibiting the mTOR/PI3K signalling pathway, thereby alleviating mitochondrial dysfunction, promoting the differentiation of BMSCs from adipogenic to osteogenic and inhibiting senescence.^273^

Huang et al.^274^ reported that mTORC1 prevents pre‐osteoblast differentiation through the Notch signalling pathway. The inactivation of mTORC1 by deleting of regulatory protein associated with mTOR (Raptor) in pre‐osteoblasts (ΔRaptor) caused lower bone mass in young mice.^275^ Subsequent studies found that mTORC1 activation can accelerate senescence in the pre‐osteoblasts, while simultaneously stimulating osteoclast formation, thereby aggravating age‐related bone loss. Reverse tests have found that inhibition of mTORC1 enables allowed pre‐osteoblasts to escape from senescence and prevented bone loss in mice. In addition, sodium voltage‐gated channel alpha subunit 1 (SCN1A) accelerates the senescence process of pre‐osteoblasts by causing depolarisation of the plasma membrane. The expression of SCN1A is positively regulated by mTORC1.^276^ The DDR signalling pathway can activate the mTOR signalling pathway in response to various forms of DNA damage.^277^ Inflammation is also a trigger for the mTOR signalling cascade, with the NF‐kB signalling pathway, a key regulator of inflammation, interacting with the mTOR pathway.^278^

The mTOR signalling pathway plays a crucial role in osteoblast senescence and is associated with various pathways and targets related to senescence.^279^ As studies on the relationship between mTOR, senescence and osteoblasts have continue, our understanding of mTOR deepens. However, the underlying mechanisms still require further elucidation.

### 4.2 p53/p21 signalling pathway

The P53 gene, a vital tumour suppressor, helps maintain genomic stability by triggering cell cycle arrest and apoptosis in response to various stressors. When a cell is exposed to stress, the p53 pathway is activated, leading to apoptosis, cellular senescence or cell cycle arrest.^280^ P21 inhibits cell growth by inhibiting CDKs, blocking the phosphorylation of Rb and E2f transcription factor (E2F).^281^ Almost all cellular senescence involves changes in p53. Studies have shown that mouse models with p53 gene knock‐in exhibit clear signs of ageing.^282^ Besides regulating senescence, p53 is a key negative regulator of osteogenesis, regulating bone remodelling by inhibiting osteoblast differentiation and promoting osteoblast‐dependent osteoclast differentiation.^283^ Loss of p53 may promote the osteogenic differentiation of BMSCs.^284^ However, other studies have shown that inhibition of p53 expression suppresses BMP2‐induced osteoblast lineage transition.^285^ It has been reported that p53 can both promote and inhibit senescence, possibly by regulating ROS.^286^ Given the important role of p53 in cell metabolism and the existence of controversial findings, research on the role of p53 has been a significant focus in the fields of ageing and osteogenesis in recent years.

Earlier studies showed increased p53 activity and decreased RUNX2 levels in murine double minute 2 (Mdm2)‐null osteoblast progenitors.^287^ It was demonstrated that the negative regulation of p53 by Mdm2 is required for osteoblast differentiation. Jin et al.^288^ found that Bre is highly up‐regulated during osteogenic differentiation. Knockdown of Bre in mouse BMSCs significantly reduced the ALP activity, expression of osteogenic genes and mineralisation ability and led to the activation of p53 signalling pathways, with manifestations of increased levels of p53, Mdm2 and p21. They found that Bre promotes Mdm2‐mediated ubiquitination and degradation of p53 through physical interactions with p53.^288^ Choi et al.^289^ found that low‐molecular‐weight protamine–superoxide dismutase 1 conjugates abolished hydrogen peroxide (H_2_O_2_)‐induced p53 and p21 activation in human dental pulp stem cells (DPSCs), alleviating oxidative stress‐induced senescence of DPSCs, and restored their osteogenic differentiation ability. Chen et al.^276^ found that plasma membrane depolarisation increases Ca^2+^ influx into pre‐osteoblasts and activates nuclear factor of activated T‐cells/activating transcription factor 3 (ATF3)/p53 signalling, thereby inducing pre‐osteoblast senescence.

Additionally, as a transcription factor, p53 can induce the transcription of various microRNAs. There is evidence that the p53/miR‐145a axis promotes the senescence of BMSCs and inhibits their differentiation into osteoblasts.^290^ Moreover, p53 is a transcriptional activator of many bone‐specific genes. A recent study using gene array analysis showed that comparing undifferentiated and differentiated osteoblasts with those lacking p53 expression revealed p53‐specific changes in microRNA expression. Overexpression of miR‐34b and miR‐140 inhibited osteoblast proliferation, and their targets included RUNX2, β‐catenin, Col1, osterix (OSX) and Wnt/β‐catenin signalling pathways.^291^ Many osteoblast‐specific genes, such as OPN, VDR, OCN, BMP2, BMP4 and BMP9, have been shown to be direct targets of p53.^291^, ^292^

The p53 signalling pathway plays a crucial role in both osteoblast precursors and osteoblast senescence. It regulates the proliferation, differentiation and functional activity of osteoblasts by affecting several key biological processes, such as the apoptosis, autophagy and cell cycle. Although continuous reports have been made on the relationship between p53 and both pre‐osteoblasts and osteoblasts in the context of ageing, the underlying mechanisms linking p53 to both pre‐osteoblasts and ageing still require extensive and in‐depth study.

### 4.3 Forkhead boxO signalling pathway

The Forkhead boxO (FOXO) family of transcription factors is crucial for regulating the genes involved in a variety of cellular physiological processes, including glucose metabolism, cellular ageing, cell‐cycle progression, programmed cell death and resistance to oxidative stress.^293^ The activity of FOXO transcription factors is also regulated by TNF‐α, lipopolysaccharide (LPS) and interactions with protein chaperones.^294^ The forkhead transcription factor family includes four members: FOXO3 (FKHRL1), FOXO1 (FKHR), FOXO6 and FOXO4 (AFX).^295^ FOXO expression varies across different tissues and cell types. FOXO3, FOXO1 and FOXO4 are expressed in bone cells,^293^ and recently, FOXO6 has also been found to be expressed in bone tissue.^296^ Current studies have found that FOXOs exert bidirectional regulation on BMSCs and osteoblasts.

#### 4.3.1 Positive regulatory effect of FOXOs on osteogenic lineage

Overexpression of FOXO1 or FOXO3 can significantly increase the expression of osteogenic markers such as OCN, RUNX2 and ALP promoting bone formation, while silencing FOXO1 can inhibit the expression of osteogenic markers and inhibit bone formation.^297^ Ageing increases oxidative stress and leads to osteoblast apoptosis, thereby reducing bone mass. Furthermore, FOXO transcription factors protect against oxidative stress by up‐regulating mitochondrial function, regulating antioxidant enzymes (catalase and superoxide dismutase) and activating free radical scavenging and apoptosis‐related genes,^298^ thereby stimulating the differentiation of BMSCs into osteoblasts and inhibiting their senescence.^294^, ^299^, ^300^ Overexpression of the FOXO3 gene in mature osteoblasts can reduce oxidative stress and further reduce the phosphorylation of p66Shc, thus inhibiting the apoptosis of osteoblasts, increasing the number of osteoblasts.^301^ FOXO1 specifically enhances the proliferation of osteoblast precursors by ATF4.^302^ Boosting SIRT1/FOXO3a signalling specifically can significantly mitigate bone loss due to 1,25‐dihydroxyvitamin D (1,25(OH)₂D) deficiency.^303^

Autophagy plays a crucial role in sustaining cellular homeostasis and is integral to a spectrum of biological processes. Jiang et al.,^304^ through both in vivo—using osteoblast‐specific Foxo1 knockout (Foxo1_OB_^−/−^, KO) and FOXO1‐overexpressing mice—and in vitro assays with primary osteoblasts, demonstrated that FOXO1 overexpression promotes bone formation and augments osteoblast function, encompassing proliferation, migration and differentiation. Furthermore, elevated FOXO1 levels triggered autophagy and strengthened the interaction with ATG7. In contrast, in 3‐month‐old mice, conditional deletion of FOXO1, FOXO3 and FOXO4 resulted in increased oxidative stress and osteoblast apoptosis in bone, as well as decreased bone formation rate, osteoblast number and bone mass in trabecular and cortical sites.^301^ It is well known that adipogenic differentiation and osteogenic differentiation are two antagonistic processes, and FOXO1 can inhibit the expression of PPARγ in BMSCs to suppress adipogenic differentiation while promoting osteogenic differentiation.^305^ These results suggest that FOXOs positively regulate osteoblast differentiation and proliferation.

#### 4.3.2 Negative regulatory effect of FOXOs on osteogenic lineage

On the other hand, FOXOs also have the effect of inhibiting osteogenesis. ROS can activate FOXO transcription factors, enhancing their interaction with β‐catenin, which in turn can reduce osteoblast generation.^306^, ^307^ Kim et al.^71^ found that decreased NAD+ levels and decreased osteogenesis in aged mouse osteoblast progenitor cells were associated with increased acetylation of FOXO1 and markers of cellular senescence. Enhancer‐mediated FOXO3 promotes the expression of lipogenic transcription factors PPARγ, C/EBP‐α and C/EBP‐β and activates the PI3K–AKT pathway to promote lipogenic differentiation and inhibit the osteogenic differentiation of BMSCs.^308^ FOXO activity is mainly regulated by the post‐translational modifications (ubiquitination, acetylation and phosphorylation) and PI3K/Akt pathway.^309^ When the PI3K/Akt signalling pathway inhibits FOXO activation, it prevents FOXO translocation to the nucleus. Conversely, when the PI3K/Akt pathway is inhibited, FOXO transcription factors can translocate to the nucleus and bind to DNA, acting as potent transcriptional activators.^310^

FOXOs have been found to attenuate Wnt/β‐catenin signalling and reduce osteoblast generation in vitro by diverting β‐catenin away from T cell factor/lymphoid enhancer factor and towards FOXO‐mediated transcription.^311^ Up‐regulation of FOXO1 in osteoblasts promoted their conversion to fat, which weakened bone mass.^294^ Interestingly, FOXO1, FOXO3 and FOXO4 were also found to inhibit the osteogenic differentiation of MC3T3‐E1 cells.^312^

Increasing evidence suggests that FOXO members interact with multiple regulators and signalling pathways, including RUNX2, ALP, ATF4 and β‐catenin, as well as those related to ageing. These interactions lead to bidirectional regulation of osteogenic processes, either enhancing or inhibiting them, throughout the stages of osteogenic differentiation. In general, the FOXO family is closely related to osteoblast differentiation. In osteoblast precursors, FOXOs are shown to inhibit osteogenesis, whereas in osteoblasts, FOXOs are shown to promote osteogenesis. This opposite effect in different cell types has piqued the interest of researchers and attracts further exploration.

### 4.4 The Yes‐associated protein (also called YAP1)/Hippo signalling pathway

The Hippo signalling pathway is a growth inhibition signalling pathway, that regulates the biological processes of cell proliferation, differentiation and senescence. Its biological effects include regulating organ size, tissue regeneration and maintaining the balance of cell proliferation and apoptosis. The Hippo pathway is regulated by mechanical environment, G protein‐coupled receptors, cell energy levels, oxidative stress, hypoxia and other signals.^313^

The core of the Hippo signalling pathway is to regulate the activity of two transcriptional coactivators, Yes‐associated protein (YAP) and PDZ‑binding motif (TAZ), through a series of cascade kinase reactions. When the pathway is not activated, TAZ and YAP are located in the nucleus and bind to transcriptional enhanced associate domain (TEAD) transcription factors to promote the expression of target genes. When the Hippo pathway is activated, mammalian sterile 20‐like kinase 1/2 (MST1/2) and large tumour suppressor homolog kinases 1/2 (LATS1/2) phosphorylate YAP/TAZ, causing them to bind to 14‐3‐3 proteins and be sequestered in the cytoplasm, thereby inhibiting their transcriptional co‐activation activity.^314^

YAP inhibits human hMSCs senescence by up‐regulating the transcription of FOXD1. Conditional deletion of YAP in h‐PDLSCs inhibited their proliferative activity and induced apoptosis, involving the Hippo pathway, and crosstalk between extracellular regulated protein kinases (ERK) and the B‐cell lymphoma‐2 (Bcl‐2) signalling pathway.^315^, ^316^ Activated YAP promoted the proliferation of h‐PDLSCs, inhibited apoptosis and delayed senescence. Additionally, it has been found that the mechanism of YAP promoting h‐PDLSCs osteogenesis in vitro and inhibiting adipogenic differentiation is partially mediated through the Wnt/β‐catenin signalling pathway via lipoprotein receptor‐related proteins and Dishevelled‐3.^317^ These studies suggest that YAP may be a candidate regulatory target for promoting h‐PDLSCs in bone regeneration.

YAP and TAZ are two homologous transcriptional coactivators that serve as key downstream effector molecules in the Hippo signalling pathway. They function to regulate cell proliferation, differentiation and the expression of inflammatory factors, being activated by both internal and external cellular signals.^318^ In the nucleus, YAP/TAZ regulate downstream gene expression by binding to and interacting with DNA from the TEAD family.^319^ The Hippo pathway negatively regulates cell proliferation and tissue growth in response to factors such as cell density, DNA damage and hormone signalling.^320^ YAP/TAZ oversee a unified genetic program tied to proliferation, encompassing genes that spur the G1/S phase transition, nucleotide mitosis, DNA replication and repair and metabolism.^318^, ^321^ This demonstrates the remarkable capacity of YAP/TAZ to control cellular senescence and boost cell growth.

Earlier studies found that YAP1/TAZ could reverse MMP14‐induced bone loss.^322^ YAP1 is capable of binding to RUNX2 and PPARγ within the nucleus, thereby facilitating the osteogenic differentiation of MSCs while suppressing adipogenic differentiation.^323^ However, one study found that low levels of YAP promoted lipogenic differentiation and inhibited osteogenic differentiation.^324^ Similarly, Xiong et al.^325^ discovered that YAP/YAP1, to some degree, restrained the differentiation of osteoblast precursor cells and diminished the transcriptional activity of RUNX2. Conversely, in fully developed osteoblasts and osteocytes, YAP/YAP1 demonstrated a function in stimulating bone formation and curbing bone resorption.^325^ De‐phosphorylated YAP/TAZ can directly block RANKL signalling or enhance osteoprotegerin (OPG) expression, thereby inhibiting RANKL signalling and suppressing RANKL‐induced osteoclast differentiation.^326^

YAP balances the relationship between MSCs osteogenesis and adipogenesis, and the regulation of YAP/TAZ on osteoblast lineage is closely related to the differentiation stage of the cells. Further elucidation of its precise mechanism in the future is expected to identify targets for regulating osteoblast lineage differentiation. Exploring ways to enhance the osteogenic potential and anti‐senescence capabilities of MSCs by modulating the activity of the YAP/Hippo pathway may provide new strategies for the treatment of age‐related bone diseases.

### 4.5 The sirtuins (SIRTs) family

The sirtuin family, comprising seven members (SIRT1–SIRT7), constitutes a class of NAD+‐dependent deacetylases.^327^ These enzymes regulate protein function by removing acetyl groups from proteins, playing a crucial role in maintaining cellular health and combating the ageing process.^328^ The functionalities of distinct SIRT members, along with their susceptibility to fluctuations in intracellular NAD+ levels, are influenced by their varying subcellular localisations. SIRT1–SIRT6 are pivotal in maintaining MQC.^329^ Zhang et al.^330^ found that SIRT1, SIRT3 and SIRT6 ameliorate osteoporosis by regulating MQC mechanisms to enhance mitochondrial protein homeostasis, biogenesis and mitophagy.

SIRT1 possesses a multitude of functions within the cell, including the regulation of gene expression, metabolism, stress responses and the control of the cell cycle.^331^ Previous studies have confirmed that SIRT1 acts as a modulator of bone mass and is positively correlated with bone metabolism and bone mass.^332^, ^333^ In recent years, a growing body of research has illuminated the pivotal role that SIRT1 plays in combating ageing.^330^, ^334^ Sun et al.^299^ found that SIRT1 overexpression in MSCs reduced the acetylation level of FOXO3a and oxidative stress, increased the expression levels of superoxide dismutase 2 (SOD2) and FOXO3a in bone tissue, enhanced the osteogenic effect and inhibited the senescence of osteoblasts, SITR3 also has a similar function. It deacetylates FOXO3, which in turn activates manganese‐containing superoxide dismutase to inhibit the production of ROS. Additionally, SIRT3 enhances mitochondrial autophagy either directly or through the PTEN‐induced kinase 1/Parkin axis, ultimately helping to remove damaged mitochondria.^335^

In aged mice, BMSCs exhibit reduced osteoblastic differentiation and increased adipocyte formation. The augmented adipogenesis was associated with relatively lower SIRT1 activity and decreased intracellular NAD+ concentrations.^336^ Khanh et al.^337^ discovered that the compromised expression of SIRT3 and SIRT1 triggers senescence in elderly adipose tissue‐derived MSCs (AT‐MSCs). However, it is solely SIRT1 that directly modulates the differentiation of beige adipocytes. The up‐regulation of SIRT1 represses the p53/p21 pathway, thereby stopping aged AT‐MSCs from transitioning into the senescent phase and rejuvenating their capacity to differentiate into beige adipocytes.^337^ Nicotinamide mononucleotide can promote osteogenesis and reduce adipogenesis by up‐regulating SIRT1 in aged bone marrow.^338^ SIRT1 can deacetylate p53, thereby inhibiting the transcriptional activity of p53, and it can also affect downstream pathways related to tissue homeostasis and diseases.^339^ This indicates that SIRT1 can regulate ageing‐related bone metabolism not only by its anti‐ROS and anti‐senescence effects but also by promoting the osteogenic differentiation and inhibiting the adipogenic differentiation of stem cells.

SIRT1 can promote osteogenic function through anti‐inflammatory pathways. Chen et al.^340^ found that Vitamin K2 can activate AMPK/SIRT1 signalling, inhibit ferroptosis in BMSCs under high glucose conditions and correct bone loss. During the differentiation of mouse ESCs into osteoblasts, blocking the action of SIRT1 and insulin resulted in a 60% decrease in osteoblast production and a 195% increase in adipocyte differentiation,^341^ suggesting that SIRT1 and insulin‐sensitive pathways may have a synergistic effect. SIRT1 has also been shown to improve age‐related senescence of MSCs by regulating telomere protective proteins.^342^ SIRT1 has been found to regulate osteoblast senescence through the acetylation of SOD2 and mitochondrial dysfunction.^343^ miR‐22 is a widely expressed microRNA, and SIRT1 has been shown to be a target of miR‐22 in MC3T3‐E1 cells. Research indicates that the up‐regulation of miR‐22 contributes to dexamethasone‐induced senescence and injury in MC3T3‐E1 cells by targeting SIRT1. Sodium hydrosulfide can prevent this process, inhibit the expression of p21 and p53 and promote the osteogenic differentiation of MC3T3‐E1 cells.^344^ The findings of these studies highlight the importance of SIRT1 in maintaining age‐related bone homeostasis through various cellular mechanisms.

When SIRT3 was knocked down, both osteogenic differentiation and of adipogenesis BMSCs were significantly reduced. However, when SIRT3 was overexpressed, the differentiation ability and longevity of senescent BMSCs (generation 7) were rejuvenated.^345^ This suggests that up‐regulation of SIRT3 can alleviate the senescence‐related defects of BMSCs. SIRT3 can reduce mitochondrial oxidative stress and mtDNA damage in osteoblasts in vitro, and SIRT3 knockout mice exhibit early‐stage osteoporosis.^346^, ^347^ Guo et al.^348^ reported that overexpression of SIRT3 significantly alleviated senescence in BMSCs induced by advanced glycation end products, improved mitophagy and ameliorated osteoporosis in ageing‐accelerated mouse strain P6 (SAMP6) mice. SIRT3 may also partially protect senescent BMSCs by modulating mitochondrial function and stabilising heterochromatin.^349^, ^350^ However, there are also contrary reports. Ho et al.^351^ designed a mouse model of SIRT3 overexpression and found that in 13‐month‐old males, the number of adipocytes in tibia slices was significantly increased. This complex phenomenon suggests that SIRT3 may affect the biological behaviour of senescence‐related osteoblasts through multiple pathways and play a dual role in osteoporosis, but more in‐depth studies are still required to clarify the mechanisms.

Earlier studies revealed that SIRT6^−/−^ mice exhibit a premature ageing phenotype and genomic instability.^352^ Recent studies found that the osteogenic ability of BMSCs was reduced after SIRT6 inhibition in vitro. Correspondingly, and in vivo study found that SIRT6 expression in BMSCs of ageing mice was decreased, bone mass was decreased and osteogenic function in bone tissue was reduced.^353^ The proliferation and differentiation of SIRT6‐knockout osteoblasts were inhibited, SIRT6‐knockout mice had reduced bone mass in vivo, and SIRT6 was found to be positively correlated with bone mass and oestrogen receptor α in human bone marrow.^354^

Together, these findings suggest the importance of SIRT proteins in osteoblast and stem cell differentiation, especially in the context of ageing‐related diseases. Further studies may lead to new therapeutic strategies for the prevention and treatment of age‐related osteoblastic senescence.

### 4.6 AMPK signalling pathway

AMPK is a heterotrimeric complex made up of three subunits: α, β and γ.^355^ The α subunit serves as the catalytic subunit, while the β and γ subunits are regulatory subunits. The activity of AMPK is regulated by the AMP/ATP ratio.^356^ When the intracellular AMP level increases, AMPK activity is activated, which in turn participates in the regulation of cellular metabolism and energy balance. AMPK can maintain the number and function of mitochondria by regulating processes such as mitochondrial biosynthesis, fission and autophagy. Concurrently, it can also enhance the antioxidant capacity of mitochondria and reduce the damage to mitochondria caused by oxidative stress.^357^

In terms of ageing, AMPK can inhibit cellular senescence through a variety of pathways. First, AMPK maintains the balance of energy metabolism and provides a sufficient energy supply for cells by regulating metabolic pathways such as fatty acid oxidation and glycolysis. Second, AMPK promotes autophagy, removing damaged organelles and proteins and preventing the accumulation of harmful substances within cells. In addition, AMPK can also reduce oxidative stress and inflammation, thereby reducing cell damage. These effects synergise to inhibit the process of cellular senescence.^358^

In terms of regulating the cell cycle and apoptosis, AMPK can inhibit the progression of the cell cycle and keep cells in the G1 or G2 phase, thereby prolonging cell lifespan.^359^ On the other hand, AMPK can promote the apoptotic process and remove damaged cells, preventing their accumulation in the body and the triggering of an inflammatory response.^360^ Ye et al.^361^ found that after activating the AMPK‐α signal, the expression of phosphorylated AMPK‐α increased, which promoted the polarisation of macrophages towards the M2 phenotype and improved the osteogenesis ability of MC3T3‐E1 cells. Melatonin is expected to enhance the osteogenic effect of hMSCs from osteoporosis patients by activating the AMPK pathway to up‐regulate FOXO3a and RUNX2.^362^ Gao et al.^363^ identified phorbol‐12‐myristate‐13‐acetate induced protein 1 (PMAIP1) as a potential risk factor for osteoporosis using gene set enrichment analysis (GSEA) and found that autophagy in osteoblasts was significantly inhibited through the AMPK/mTOR pathway. Concurrently, si‐PMAIP1 can up‐regulate the expression of p‐AMPK and down‐regulate the expression of p‐mTOR to promote the proliferation and differentiation of osteoblasts and inhibit osteoporosis.^363^ AMPK and mTOR have an antagonistic relationship. When AMPK is activated, it inhibits mTOR, which is a key mechanism that shifts cells from anabolic (accumulation) to catabolic (decomposition) processes. Conversely, active mTOR can suppress AMPK signalling through multiple feedback mechanisms, thereby maintaining metabolic homeostasis.^364^

In addition, AMPK also inhibits ageing‐related bone loss by inhibiting the replicative senescence of stem cells, enhancing osteoblast autophagy, inhibiting adipogenic differentiation and up‐regulating osteogenesis‐related factors.^365^, ^366^ The AMPK signalling pathway plays a key role in regulating osteoblast senescence and the development of osteoporosis. Further understanding of AMPK regulatory mechanisms is important for developing new therapeutic strategies.

### 4.7 Insulin/IGF‐1 signalling pathway

The IGF‐1 pathway shares similarities with the insulin signalling pathway and is considered one of the most evolutionarily conserved ageing pathways.^367^ IGF‐1, as a polypeptide hormone, binds to its homologous IGF‐1 receptor (IGF‐1R, α subunit) on target cells, triggering the autophosphorylation of its tyrosine kinase domain. This further activates the kinase activity of IGF‐1R, which in turn activates the downstream signal transduction pathway. This activation then regulates cell growth, differentiation and metabolism.^368^ The IGF‐1 pathway involves the activation of downstream signalling cascades and interactions between various signalling pathways, such as the PI3K/AKT, Janus kinase/signal transducers and activators of transcription (JAK/STAT), MAPK and mTOR pathways. The activation and crosstalk of these signalling cascades lead to changes in gene expression that promote cellular stress resistance, reduce inflammation, improve metabolic efficiency and further regulate ageing‐related processes.^369^, ^370^ In the context of bone health, ageing has been shown to affect osteoblasts by altering not only the IGF‐1 signalling pathway but also other pathways, such as the Wnt and Hedgehog pathways.

During the 1990s, mice with IGF‐1 or IGF‐1R knockouts were extensively studied.^371^ IGF‐1R activation on MSCs triggers a signalling cascade through the PI3K–Akt pathway, leading to the activation of mTOR, elevated expression of RUNX2 and OSX and promoting osteoblast differentiation.^372^ In pre‐osteoblasts, IGF‐1R activates and promotes Col1 synthesis via OSX, and OCN secretion and ALP activity are elevated.^373^ Crane et al.^374^ observed that bone volume and trabecular bone thickness were decreased in an inducible IGF‐1R Cre/lox knockout mouse model. Exendin‐4, a glucagon‐like peptide 1 receptor agonist, has been shown to enhance the proliferation of senescent osteoblasts by activating the IGF‐1/IGF‐1R signalling pathway.^375^ Activation of the ERK pathway and the IGF‐1/IGF‐1R signalling pathway plays a critical role in osteoblast function and bone remodelling, and lactoferrin has shown potential to improve age‐related osteogenic inhibition.^376^ This highlights the importance of the Insulin/IGF‐1 signalling in the differentiation, proliferation and function of osteoblasts.

IGF‐binding proteins (IGFBPs) have been identified as an important component of the IGF signalling pathway, and IGFBPs bind to IGFs to regulate their activity.^377^ Other kinases, such as Src and focal adhesion kinase (FAK), also phosphorylate and activate IGF‐1R.^378^ The role of telomerase activity in promoting osteoblast differentiation by regulating the IGF signalling pathway has been highlighted in several studies.^379^, ^380^ Both IGFBP‐3 and IGFBP‐4 can induce cell senescence and impair the differentiation potential of osteoblasts; telomerase activity can counteract this effect by regulating the IGF signalling pathway to promote osteoblast differentiation.^381^ Intervention with AG1024 (Tyrphostin AG 1024), a blocker of IGF‐IR, can reverse IGFBP‐4‐induced proliferation inhibition.^382^ Knocking out IGFBP‐4 stimulates the activation of ERK and Smad by increasing phosphorylation and restores the osteogenic potential of elderly MSCs.^383^ Pregnancy‐associated plasma protein‐A (PAPP‐A) is a metalloproteinase that cleaves IGFBPs, specifically IGFBP‐2, IGFBP‐4 and IGFBP‐5, to increase the availability of IGFs.^384^ In human dental pulp cells, the expression of IGF‐2 was increased under osteogenic conditions, the cleavage of IGFBP‐4 was increased and the expression of stanniocalcin‐2 (STC2) was decreased.^385^ Mohrin et al.^386^ found that PAPP‐A is expressed by MSCs in multiple tissues and enhances IGF signalling in these cells, while anti‐PAPP‐A can significantly reduce local IGFBP‐5 in mouse bone marrow. Subsequently, IGF signal transduction in MSCs is inhibited, the expression of ECM genes is down‐regulated, and the number of osteoblasts and bone mineralisation are reduced.^386^ Dysregulation of ECM production is associated with ageing and leads to age‐related diseases. IGFBP‐7 partially facilitates the osteogenic differentiation of BMSCs via the Wnt/β‐catenin signalling pathway and boosts the expression of osteogenic‐specific genes.^387^ Lu et al.^388^ found that IGFBP‐7 can partially reprogram human fibroblasts into osteoblast‐like cells through mechanisms that induce a senescence phenotype and autocrine IL‐6 signalling. Therefore, IGFBP‐5 and IGFBP‐7 are considered to have a positive regulatory effect on osteogenic function.

IGF‐1, which is secreted by osteoclasts, has been reported to promote osteoblast differentiation and play a crucial role in bone formation.^389^ On the other hand, osteoblasts with insulin receptor deficiency exhibit impaired differentiation and maturation, decreased RUNX2 expression, reduced bone mass and significant peripheral obesity and insulin resistance.^390^

The Insulin/IGF‐1 signalling pathway plays a central regulatory role in the ageing processes and physiological processes of stem cells and osteoblasts. Thus, gaining a comprehensive insight into the regulatory mechanisms of the Insulin/IGF‐1 signalling pathway holds immense importance for devising interventions aimed at bone diseases stemming from senescence in osteoblast lineages.

### 4.8 Hedgehog signalling pathway

The components of the Hedgehog signalling pathway mainly include the suppressor of fused, Hh ligand, smoothened receptor (Smo), Patched receptor (Ptch) and the transcription factor glioma‐associated oncogene (Gli). The Hedgehog protein family includes Desert Hedgehog, Indian Hedgehog (Ihh) and Sonic Hedgehog (Shh).^391^ Gli1, Gli2 and Gli3 serve as the principal downstream effectors of the Hedgehog signalling pathway. They function as nuclear transcription factors, binding to promoters to modulate the expression of target genes. Al‐Azab et al.^392^ found that BMSCs transfected with Ihh siRNA exhibited characteristics of age‐related features, including increased SA‐β‐gal activity, induction of cell cycle inhibitors (p53/p16), development of the SASP, activation of mTOR and ROS pathways and promotion of biased differentiation. In mice lacking Ihh, osteoblast differentiation was impaired, and RUNX2 expression was insufficient.^393^ H_2_O_2_ is commonly used as an inducer in oxidative stress‐induced cell senescence models. Exogenous H_2_O_2_ can inhibit Shh‐mediated osteogenic differentiation of mice and other MSCs lines in vitro and decrease the expression of ALP, OSX and bone sialoprotein.^394^

In the presence of osteogenic stimuli, MSCs express receptors for Hh ligands, including Ptch and Smo. When Hh proteins bind to Ptch, they relieve the inhibition of Smo, triggering a cascade of intracellular signalling events that ultimately leads to the activation of Gli transcription factors.^314^ Gli2 and Gli3 are the main effectors of Hedgehog signalling in osteoblasts.^395^ They enter the nucleus and regulate the expression of osteogenic genes, including those encoding proteins involved in bone matrix synthesis (e.g., Col1, OCN), bone mineralisation (e.g., ALP) and osteoblast differentiation (e.g., RUNX2). By up‐regulating these genes, Hedgehog signalling promotes the differentiation of MSCs into mature osteoblasts and enhances their ability to form bone. Furthermore, Hedgehog signalling may also influence osteoblast proliferation indirectly by regulating the expression of growth factors such as IGF and BMPs.

Inhibition of Laminin α2 has likewise been demonstrated to boost the osteogenic differentiation and curb the adipogenic differentiation of MSCs by regulating the Hedgehog pathway.^396^ NTRK‐like protein‐5 (Slitrk5) and SLIT have been shown to be negative regulators of Hedgehog signalling in osteoblasts, and the loss of Slitrk5 enhances osteoblast differentiation in vitro and in vivo.^397^ As mentioned earlier, Hedgehog signalling inhibits the differentiation of BMSCs into adipocytes and promotes their differentiation into chondrocytes and osteoblasts.^398^ The Hedgehog pathway also decreases with ageing, and a decreased ability of BMSCs to secrete RUNX2 ultimately leads to a decreased osteogenic ability of BMSCs.^261^ However, other studies suggest that Hedgehog signalling reduces osteoblast differentiation of hMSCs.^399^ Recent studies have suggested that Hedgehog signalling may modulate the response of osteoblasts to mechanical stimuli, thereby influencing bone adaptation and remodelling.^400^

These studies suggest that Hedgehog signalling is closely linked to the osteoblast lineage associated with senescence and may be a potential therapeutic target for diseases related to the osteoblast lineage.

### 4.9 Other signalling pathways involved in osteoblast senescence

The signalling pathways described above are not exhaustive in regulating the mechanisms associated with senescence in osteoblast lineages. Various intracellular signalling pathways also regulate the senescence of osteoblast lineages directly or indirectly. Below, we provide a general introduction to some additional signalling pathways.

#### 4.9.1 P16/RB signalling pathway

It is characterised by p16, a tumour suppressor gene. Since its discovery, the importance of p16 in the field of ageing has been increasingly recognised.^401^ P16 is a specific inhibitor of CDKs CDK6 and CDK4, primarily preventing the transition of cells from the G1 to the S phase by keeping pRB in a hypophosphorylated state, thus causing subsequent proliferative arrest.^402^ The expression of p16 significantly increases in various tissues of elderly rodents, and the removal of p16‐positive senescent cells delays the onset and progression of age‐related pathologies and extends the lifespan of both prematurely and naturally aged mice.^57^, ^403^ P16 deficiency may prevent osteoporosis induced by oestrogen deficiency by inhibiting oxidative stress, osteocyte senescence, osteoclast bone resorption and stimulating osteoblast formation.^404^ Similarly, p16 deletion has been found to enhance the differentiation and migration of BMSCs, up‐regulate the expression of p‐mTOR, hypoxia‐inducible factor (HIF)‐1α, p‐AKT and vascular endothelial growth factor (VEGF)‐A and promote osteoblast generation in older mice, as well as promote angiogenesis to accelerate fracture healing.^405^ Modifying p16 could be a novel strategy for treating bone diseases in the elderly by counteracting osteoblast senescence.

#### 4.9.2 HIF‐1α pathway

HIF‐1α promotes bone formation by regulating the differentiation and proliferation of osteoblasts and also enhances the vascularisation of bone tissue. Shomento et al.^406^ observed that mice lacking HIF‐1α in osteoblasts had reduced bone mass. HIF‐1α can activate and promote osteoblast differentiation in BMSCs both in vivo and in vitro.^407^ In a recent study, Shao et al.^408^ found that the expression of ageing markers (p53, p21 and p16) increased in the femurs of aged C57BL/6 J male mice, along with HIF‐1α. HIF‐1α and ageing markers were up‐regulated in aged BMSCs. After transfection with HIF‐1α siRNA, the down‐regulation of osteogenic and angiogenic markers (ALP, RUNX2 and VEGF) in aged BMSCs was partially reversed. Therefore, HIF‐1α has the potential to exhibit different roles and functions depending on whether the cell is senescent or not.

#### 4.9.3 HIF‐2α pathway

Lee et al.^409^ found that the expression of HIF‐2α and p21 was enhanced and osteogenic differentiation was inhibited in a mouse primary cranial pre‐osteoblast senescence model induced by doxorubicin. They further observed that HIF‐2α blocks the differentiation of pre‐osteoblasts by increasing the expression of twist family basic helix‐loop‐helix (BHLH) transcription factor 2. This action inhibits the expression of OCN and RUNX2.^410^ HIF‐2α is a negative regulator of osteoblast formation and bone mass increase.^411^

#### 4.9.4 HIF‐3α pathway

HIF‐3α is a competitor of HIF‐1α. Mir‐29cb2, which is a target of HIF‐3α,^412^ can inhibit the effect of HIF‐1 by competing with HIF‐1β subunits. This results in decreased osteoblast activity, delayed bone remodelling, reduced angiogenesis and ultimately osteoporosis.^413^ However, further clarification is needed regarding the underlying mechanisms of HIF‐3α’s role in age‐related osteogenic differentiation.

#### 4.9.5 Nrf2 pathway

Nrf2 is essential for maintenance of MSCs’ self‐renewal capacity and osteogenic differentiation. Levels and activity of Nrf2 may decrease with age.^414^ Deletion of the Nrf2 gene may lead to increased ROS, decreased biological function and accelerated cell senescence.^148^ Phosphorylation and nuclear import of Nrf2 increase SIRT1 protein levels in late‐passage BMSCs by reducing p53 protein levels, thereby maintaining the stemness of BMSCs and promoting osteogenic potential.^415^ Liu et al.^416^ found that ubiquitin binding enzyme E2E3 (UBE2E3) had high expression levels in bone marrow and was positively correlated with osteogenic genes. Down‐regulation of the UBE2E3 gene can inhibit the nuclear translocation and activity of Nrf2, accelerate cell senescence and inhibit the osteogenic differentiation of juvenile BMSCs in vitro.^416^ Casati et al.^417^ found that increasing the glutathione/oxidised glutathione ratio attenuates ROS‐induced oxidative damage in osteoblasts through the PI3K/AKT–Nrf2 signalling pathway.^418^

#### 4.9.6 NF‐κB signalling pathway

The NF‐κB signalling system is a major regulator of immune and stress responses and also initiates cellular senescence and SASP production.^419^ NF‐κB signalling can be triggered by ROS, immune responses, DNA damage and other senescence reactions.^420^ It can be enhanced by signalling pathways such as cyclic GMP‐AMP synthase—stimulator of interferon genes, high mobility group box 1 and other inflammatory mediators including NLRP3, LPS, IL‐1β and TNF‐α.^421^ ROS induce down‐regulation of peptidyl arginine deiminase 2 expression in MC3T3‐E1 cells, stimulate the production of SASP and the activation of NF‐κB, thereby accelerating senescence and osteogenic dysfunction in MC3T3‐E1 cells. Inhibition of NF‐κB by the drug inhibitor BAY11‐7082 or siRNA effectively alleviated H_2_O_2_‐induced senescence, accumulation of γH2AX foci and osteogenic dysfunction in MC3T3‐E1 cells.^212^

#### 4.9.7 Wnt/β‐catenin signalling pathway

It is involved in many physiological processes such as cell differentiation, proliferation and tissue development and is an effective inhibitor of fat formation.^422^ It can maintain bone homeostasis by promoting anti‐senescence of MSCs and inhibiting SASP.^423^ The Wnt/β‐catenin signalling pathway promotes the differentiation of osteoblast precursor cells into osteoblasts and inhibits their differentiation into adipocytes, with Wnt6, Wnt10a and Wnt10b promoting osteogenic differentiation of MSCs through this pathway.^424^ Furthermore, Wnt10b regulates osteogenesis of AdMSCs through the Wnt/β‐catenin signalling pathway, particularly in osteoporosis.^425^ The Wnt/β‐catenin signalling pathway engages in interactions with various other signalling pathways, such as Notch signalling, BMP, Hedgehog signalling and parathyroid hormone (PTH) signalling, which jointly affect the differentiation and function of osteoblasts.^426^ However, when chronically activated, this pathway may lead to abnormal function in osteoblast cells, promoting ageing.^427^ Some transcription factors or pathway antagonists, like SRY‐box transcription factor 2 (Sox2), can inhibit the Wnt/β‐catenin signalling pathway and have osteogenic effects by binding β‐catenin, keeping MSCs in an undifferentiated state and reducing osteogenic differentiation potential.^428^ Additionally, secreted Dickkopf‐1 and Frizzled‐related proteins can bind to the receptors of the Wnt/β‐catenin signalling pathway, inhibit signal transduction and affect the differentiation of osteoblasts.^427^

**FIGURE 4:**
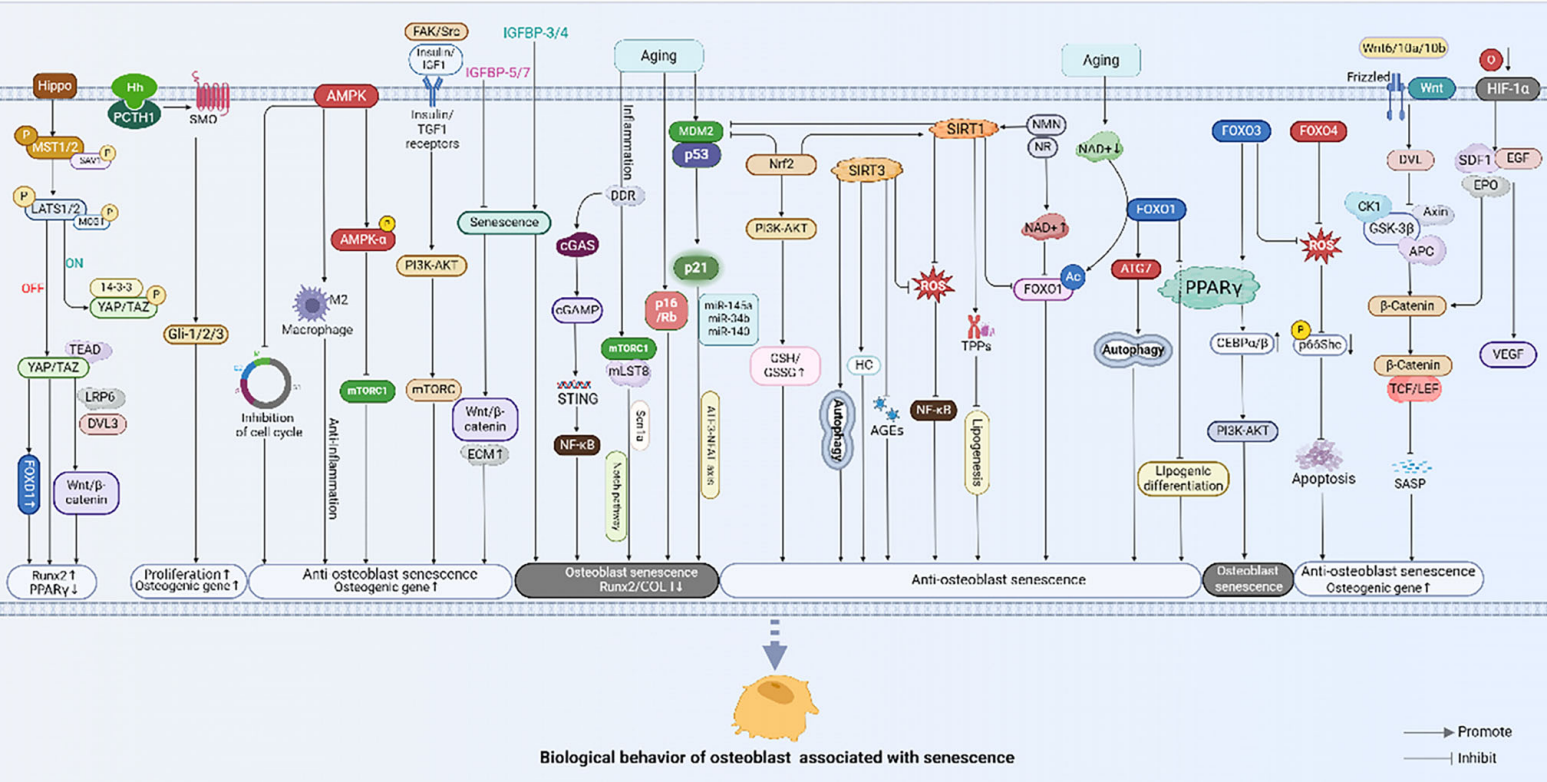
Osteoblast senescence‐related signalling pathway.

Among the many signalling pathways related to osteoblast senescence, different pathways and the expression of related proteins or genes vary depending on the environment, period (early and late osteoblast) and stimulation sources. These pathways interact in complex ways (Figure 4). A deeper understanding of these regulatory mechanisms is important for developing therapeutic strategies for bone metabolic diseases associated with senescence in osteoblast lineages. This is indeed a fascinating area that will undoubtedly inspire further exploration of the field.

## 5 STRATEGIES TO INHIBIT OR ANTAGONISE SENESCENCE IN OSTEOBLASTS

**FIGURE 5:**
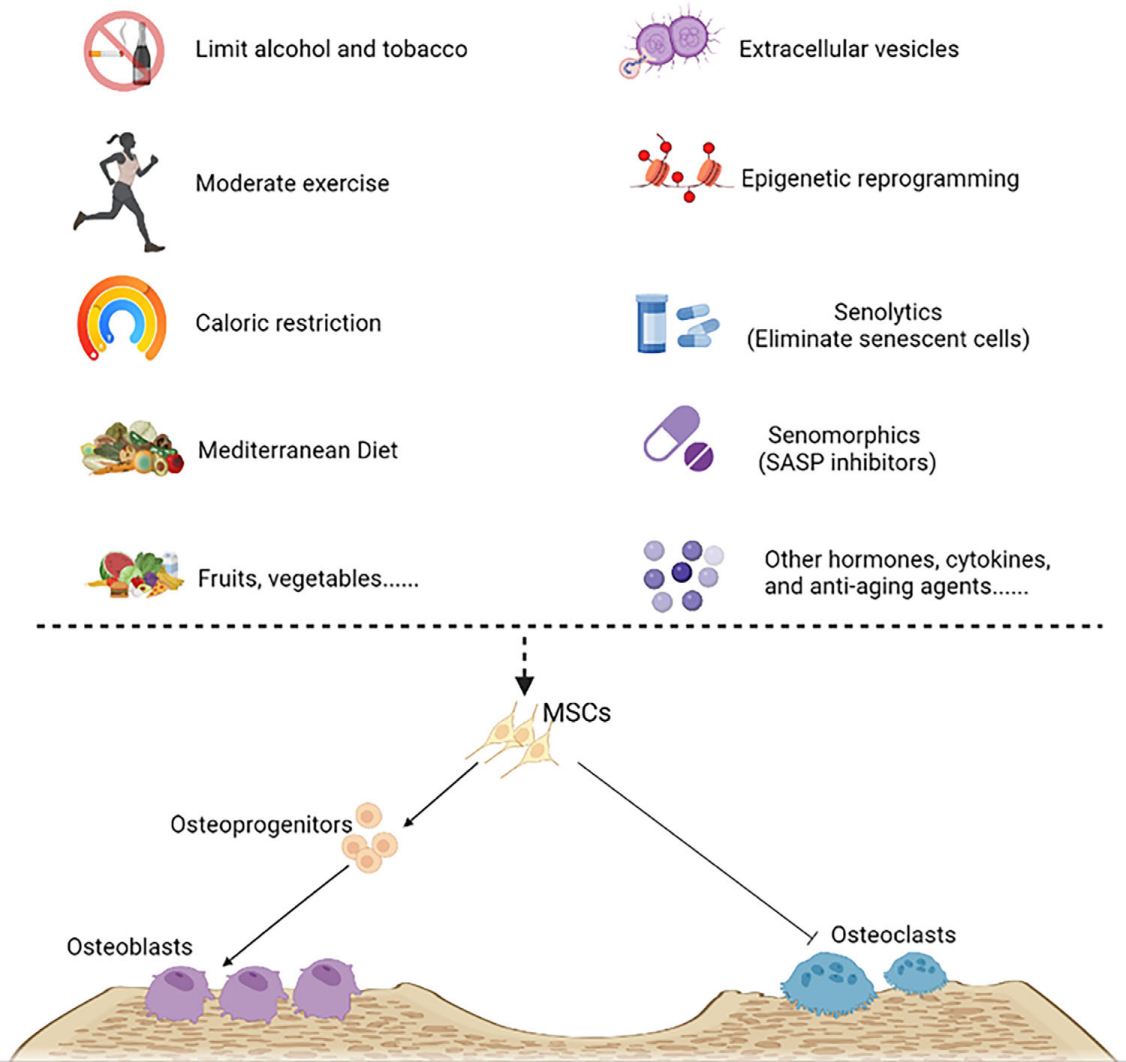
Strategies to inhibit or antagonise senescence in osteoblast.

According to previous studies on ageing, the Geroscience hypothesis posits that any anti‐ageing intervention will simultaneously delay both the onset and severity of multiple chronic diseases.^429^, ^430^ This suggests that delaying the body's ageing process or inhibiting the secretion of ageing‐related substances could alleviate or postpone age‐related chronic diseases, including those affecting osteoblast lineages. Scientists are now proposing strategies to combat ageing, aiming to improve the health and therapeutic potential of osteoblasts and to delay the onset of age‐related bone diseases (Figure 5).

### 5.1 Make nutrition and lifestyle changes

Nutritional habits are closely linked to cellular senescence. Caloric restriction (CR) is a dietary intervention where caloric intake is reduced without causing malnutrition.^431^ CR decreases the production of ROS, enhances the body's antioxidant defences and increases autophagy, thereby reducing oxidative damage to cells and DNA.^432^ CR also regulates several pathways, such as AMPK, mTOR and IGF‐1, preventing stem cell senescence and enhancing their proliferation and self‐renewal by maintaining their cellular and acellular niches.^433^ A clinical study on CR found that participants who reduced their calorie intake by about 14% over 2 years produced more T cells, had reduced levels of platelet‐activating factor acetylhydrolase and slowed down the ageing process.^434^ Studies indicate that adherence to the Mediterranean diet (Med‐Diet) can prevent osteoporosis.^435^, ^436^ Olive oil, with a high proportion of phenols, can increase the activity of ALP and the amino‐terminal propeptide of type I procollagen (P1NP, a classic osteogenic marker) and promote the production of ECM to enhance osteoblast growth.^436^, ^437^ Adhering to a healthy eating pattern that includes fruits, vegetables, whole grains, poultry, fish, nuts and legumes, while avoiding ultra‐processed foods, helps combat ageing and promotes bone health.

For smokers, nicotine in cigarettes increases the release of pro‐inflammatory mediators, leading to a state of high glucocorticoids in the body, slowing the production of osteoblasts and promoting the activity of osteoclasts.^438^, ^439^ Quitting smoking can reverse this phenomenon.^440^

Light to moderate alcohol consumption, which is a component of the Med‐Diet, does not have significant negative effects on bones. However, excessive alcohol intake can impair nutrient absorption, including reduced calcium absorption in the intestine. This can lead to decreased circulating serum calcium levels and result in calcium deficiency.^441^ Testosterone, a hormone that promotes the growth and differentiation of osteoblasts,^442^ can be adversely affected by alcohol abuse. In males, this may manifest as decreased testosterone secretion, increased acetaldehyde levels and reduced OPG levels, potentially causing osteogenic dysplasia.^443^ In women, heavy drinking can reduce oestrogen production, leading to diminished bone formation.^444^ In vitro studies have shown that osteoblasts treated with excessive ethanol may undergo a form of programmed necroptotic cell death through the up‐regulation of ROS and the receptor‐interacting protein kinase (RIPK) 1/RIPK3 pathways.^445^ While long‐term excessive alcohol consumption is detrimental to osteogenic function, the impact of moderate alcohol consumption on osteoblast differentiation and function remains a subject of debate.^444^, ^446^, ^447^

### 5.2 Moderate physical exercise

Mechanical stress from exercise can promote the osteoblast differentiation and osteogenic function, inhibit the differentiation of BMSCs into adipocytes and prevent bone mass loss.^448^ Ion channels, gap junction proteins, integrins, FAK, the ECM, primary cilia and cellular skeletal components—such as microtubules, intermediate filaments and actin filaments—serve as mechanical sensors that regulate intracellular signalling pathways.^11^ The first response of osteoblasts the mechanical stress induced by exercise involves the action of target genes through various pathways (such as Ca^2+^, ECM–integrin–cytoskeleton, Piezo1 and cell regulators). These pathways convert mechanical signals into chemical signals, regulating of various receptors on the cytoplasm, membrane and nucleus to modulate bone formation.^449^, ^450^, ^451^ In addition, physical exercise can induce MSCs migration and differentiation, reduce the expression of p16 and p53, counteract senescence‐related inflammation, delay telomere shortening and increase DNA methylation.^452^ Aerobic exercise may reduce inflammation by decreasing the release of pro‐inflammatory cytokines IL‐18 and IL‐1β.^453^ Moreover, physical exercise can activate the PTH and Wnt signalling pathways, positively influencing osteoblast formation.^454^, ^455^ A clinical study from NCT04815824 found that 38 elderly veterans (aged 60 years and above) completed both cycling and treadmill walking exercises and observed the acute effects on bone metabolism markers 48 h after the exercise. It was found that weight‐bearing exercises (treadmill walking) cause less disruption to calcium homeostasis and less activation of bone resorption compared with non‐weight‐bearing exercises (cycling).^456^ However, different types of exercise have varying effects on bone metabolism and bone formation, allowing individuals to customise their exercise routines to promote bone metabolism and maintain bone health.

### 5.3 Extracellular vesicles

EVs are small vesicles enclosed by membranes that cells release. They are usually found in the ECM, different bodily fluids, or the liquid left over from cell cultures. Depending on how they are made, their size and what they look like, they can be sorted into three main kinds: exosomes (30–150 nm wide), microvesicles (100–1000 nm wide) and apoptotic bodies (1–5 µm wide).^457^ Exosomes convey biological signals to modulate the biology of target cells, participating in both physiological and pathological processes. They carry a variety of components, including proteins, lipids, drugs, nucleic acids (mRNA, DNA and non‐coding RNA), metabolites and amino acids, facilitating intercellular communication.^458^

Recent studies have shown that exosomes carrying miR‐27a‐5p, miR‐21‐5p, miR‐221, miR‐935 and miR‐151‐5p may effectively enhance osteoblast differentiation and alleviate osteoporosis.^459^, ^460^ Meng et al.^461^ found that young plasma exosomes can significantly enhance the proliferation, migration and osteogenic differentiation of BMSCs through miR‐217‐5p in vitro, effectively alleviating osteoporosis symptoms in mice. EVs can also be combined with biomaterials to influence the biological behaviour of osteoblast lineages. For example, Han et al.^462^ 3D bioprinted periodontal cell‐derived small EVs in 10% gelatin methacryloyl using microextrusion bioprinting and performed a 2‐week intervention on hBFP‐MSCs, which enhanced calcium formation, ALP staining and toluidine blue staining. However, not all EVs have positive effects on promoting osteoblast differentiation and proliferation. For instance, exosome containing miR‐424‐5p can inhibit the Wnt inhibitory factor 1/Wnt/β‐catenin‐mediated activity of OPN, ALP, OCN and RUNX2, thereby preventing osteoblast development.^463^ EVs derived from different cells and tissues have diverse functions and effects, playing various roles in ageing, proliferation and differentiation of osteoblasts.^464^ In some cases, MSCs‐derived EVs also promote osteogenesis by enhancing vascularisation, increasing macrophage M2 polarisation, decreasing macrophage M1 polarisation and reducing pro‐inflammatory factor expression.^465^, ^466^

Another study found that osteoclast‐derived EVs with a particle size less than 100 nm inhibited osteoblast differentiation, while EVs larger than 100 nm induced osteoblast differentiation.^467^ Generally, regulating the expression of cargos in EVs via plasmids or electroporation, or adding specific peptide modifications to the EVs surface, or loading EVs onto biomaterial scaffolds, can improve the therapeutic efficiency of EVs.^468^ A recent study has revealed that small EVs derived from the plasma of young mice can combat pre‐existing ageing across cellular, physiological, mitochondrial and molecular levels.^469^ As a promising therapeutic agent, EVs are favoured by researchers for their high stability, good bioavailability and low immunogenicity in biological environments. However, further research needed to refine the selection of extraction sources, distribution of biological targets and standardised specifications to enhance their therapeutic potential. With the deepening of research on the non‐cellular origin of EVs,^470^ EVs‐based non‐cellular regenerative medicine holds promising prospects and challenges in treating senescence‐related osteoblast lineages.

Currently, the use of EVs for treating of ageing‐related osteoporosis has largely been focused on small animal models. While these models provide important preclinical references, there are differences between animal pathophysiology and human disease. Nearly 100 preclinical studies of EVs have been registered at www.clinicaltrials.gov to explore the efficacy and limitations of EVs in clinical studies. However, the standardisation of EVs production faces several challenges. These include the heterogeneity of EVs, the lack of standardised separation and characterisation methods and the difficulties in scaling up production. The heterogeneity of EVs makes it difficult to compare the results of different studies and ensure the consistency of their therapeutic effects.^471^ Different centrifugation techniques, such as ultracentrifugation and size‐exclusion chromatography, can produce EVs with different characteristics and purities.^472^ Some traditional methods for collecting EVs are time consuming and not conducive to large‐scale production. Additionally, there is a lack of unified standards for specific markers to identify and differentiate various EVs subtypes.^473^ Addressing these challenges is crucial for advancing research in the field of EVs and fully realising their therapeutic potential.

### 5.4 Mitochondrial transfer

Mitochondrial transfer is an emerging strategy to combat cellular senescence and enhance tissue regeneration. One study found that melatonin‐loaded mesoporous bioactive glass microspheres facilitate the transfer of mitochondria by forming tunnelling nanotubes, thereby enhancing mitochondrial function and improving bone regeneration in aged rats.^474^ Nuerlan et al.^475^ demonstrated that mitochondrial transfer can enhance the osteogenesis of senescent PDLSCs and facilitate bone repair in aged rats. This approach leverages the therapeutic potential of mitochondria to rejuvenate senescent cells and promote osteoblast function. By restoring mitochondrial function, this strategy holds promise for enhancing bone health and mitigating age‐related bone loss.

### 5.5 Epigenetic reprogramming

Recent studies have found that epigenetic reprogramming can reverse age‐related changes and enhance cell function. For instance, induced reprogramming can lead to genome‐wide epigenetic alterations that reverse many age‐related changes observed within cells.^476^ Scientists have been exploring the possibility of reversing cellular senescence by partially reprogramming human cells, making them more youthful.^477^ This approach, known as epigenetic reprogramming or cellular regeneration, holds promise for reducing the epigenetic age and enhancing cell function without altering the underlying genome.^476^

Xu et al.^478^ found that injecting of senescent cells into young mice led to the premature appearance of many age‐related lesions in organs far from the injection site. When senescent preadipocytes were transplanted into young mice, it led to physical dysfunction and a shortened lifespan.^478^ Conversely, transfusing the blood of young mice into old mice has given rise to the idea that ‘young blood’ may hold the potential for rejuvenation.^479^, ^480^ Studies have shown that transfusions of young mouse blood into old mice can reverse ageing and rejuvenate the recipients; the epigenetic gene expression signatures of the old mice become more similar to those of young mice, and their physical function is significantly improved.^481^ This phenomenon is believed to be due to the underlying mechanism of epigenetic reprogramming.^480^, ^482^ Furthermore, as research progresses, the interplay among blood composition, the gut microbiome and the epigenetic clock is being increasingly understood, shedding light on its role in numerous diseases and the ageing process. ^483^, ^484^ Since epigenetic changes are reversible, early identification of epigenetic marks may represent a promising breakthrough for future gerontology research.

Although epigenetic reprogramming holds promise for treating ageing‐related diseases, it also carries potential risks of carcinogenesis. This is because introducing changes to the epigenome that regulates gene expression can inadvertently activate oncogenes or silence tumour suppressor genes, ultimately leading to uncontrolled cell growth and cancer development.^485^ To reduce the risk of cancer, epigenetic reprogramming therapy needs to be highly precise, targeting specific genes and pathways without disrupting overall genomic stability or causing unintended cellular changes.

### 5.6 Cytokines and drugs treatment

To date, several studies have shown promising effects in inhibiting senescence in osteoblast lineages to promote osteogenic differentiation and cell proliferation. These include anti‐ROS measures, regulation of signalling pathways related to ageing bone formation, inhibition of SASP secretion and clearing of senescent cells.

*Senolysis*: This process involves removing senescent cells from the ecological niche of normally proliferating cells using specific reagents that selectively remove eliminate these cells. Farr et al.^486^ used a combination of senolytics (dasatinib + quercetin) to specifically eliminate senescent cells and promote bone formation and mineralisation of MC3T3‐E1 cells and AdMSCs. Given the excellent anti‐ageing effects of senolytics, clinical trials were initiated in 2016 to explore their clinical effects and determine any side effects in humans. The Hematopoietic Stem Cell Transplant Survivors Study (NCT02652052) is ongoing.^487^ The results of a 20‐week clinical trial on senescent cell clearance in elderly female subjects (NCT04313634, with a total of 67 participants reaching the experimental endpoint) showed that senolytics significantly improved bone formation, as indicated by an increase in P1NP, which was superior to both fisetin and the control group. Senolytics also potentially inhibited bone resorption in the short term, as evidenced by a decrease in C‐terminal telopeptide. The effects of senolytics and fisetin on bone mineral density were both minor; however, the senolytics group exhibited slightly less bone loss in the lumbar spine and femoral neck compared with the fisetin group. Adverse events in the senolytics group were mainly mild, such as headaches (53.3%) and diarrhoea (23.3%), with no serious adverse events reported and good overall tolerance.^488^ Although the combination of senolytics is expected to serve as a senolytic for treating age‐related diseases, it may have off‐target effects. These include potential side effects such as pulmonary oedema, thrombocytopenia and neutropenia, as well as impacts on various biological pathways due to their inhibition of tyrosine kinases and PI3K.^489^ To prevent side effects of dasatinib, a ‘hit‐and‐run’ strategy of intermittent dosing has been proposed,^490^ but its efficacy remains to be observed. Studies have shown that urokinase fibrinogen activated receptor‐specific chimeric antigen receptor (CAR)‐T cells can effectively eliminate senescent cells in vivo and in vitro,^491^ and therapeutic CAR‐T cells targeting senescent cells have been proposed as new senolytics.^492^ However, research on the effects of CAR‐T cells on bone ageing is still in the exploratory phase, yet it also holds promise for future therapeutic strategies targeting osteoporosis related to osteoblast senescence.

*SASP inhibitors (Senomorphics)*: These have shown similar effects by inhibiting the production of SASP factors.^493^ The addition of a JAK1 inhibitor can block the secretion and expression of SASP factors, such as MMP‐3, IL‐1α, adiponectin and resistin, alleviating the negative effects of radiation‐induced senescence in MLO‐Y4 cells and of SASP on BMSCs' osteogenic differentiation.^215^ However, non‐senescent cells, such as macrophages, can also secrete SASP factors and non‐specific anti‐SASP treatment may bring significant negative effects.^494^ Additionally, SASP also has a positive side; for instance, The SASP released by senescent cells can boost the reprogramming of adjacent cells. Moreover, the temporary expression of reprogramming factors can foster the regeneration of surrounding tissues and decelerate tissue ageing.^495^, ^496^ Therefore, improving the specificity of SASP inhibitors may enable more targeted anti‐ageing treatments.

Other factors, including hormones, cytokines and anti‐ageing agents, have also been shown to reverse senescence in osteoblast lineages. For example, sclerostin inhibits osteoblast activity by blocking activation of the classical Wnt signalling pathway and stimulates osteoclast generation by up‐regulating RANKL. The sclerostin inhibitor romosozumab has the opposite biological effect, inhibiting osteosclerotic hormone.^497^ Yu et al. recent studies showed that oestrogen attenuated the nuclear import of p53 and accelerated the degradation of p53 in osteocyte‐like MC3T3‐E1 and cells MLO‐Y4 cells. They suggest that oestrogen regulates ubiquitin‐specific peptidase 10 (USP10) to accelerate the degradation of p53 and inhibit its nuclear translocation, thereby preventing MC3T3 cell senescence and bone loss.^498^ BMP9 has significantly down‐regulated the expression of the DNA damage marker γH2AX in senescence‐induced MC3T3‐E1 cells, inhibited the secretion of SASP and inhibited osteoblast senescence through the SMAD family member 1 (Smad1)–STAT1–p21 axis, reducing age‐related bone loss.^499^ Bae et al.^500^ reported that melatonin could inhibit ethanol‐induced human periodontal ligament cells (hPDLCs) senescence through a cascade of AMPK, mTOR and MAPK signalling, thereby protecting hPDLCs from differentiating into osteoblasts. Gong et al.^501^ found that melatonin could prevent hyperglucose‐induced excessive ROS production and DNA damage senescence in MC3T3‐E1 cells by increasing the expression of CDK2, CDK4, RUNX2 and cyclin D1 through melatonin receptor (MT1 and MT2) pathways. Meanwhile, melatonin also reduced the overexpression of p16, γH2AX, p21, p53 and ATM in MC3T3‐E1 cells.^501^ N6‐methyladenosine (m6A) methylation, the most prevalent internal modification in eukaryotic messenger RNA, plays a crucial role in regulating various biological processes, including RNA splicing, nuclear export, translation and degradation. Methyltransferase‐like 3, a key enzyme in m6A methylation, enhanced the stability of heat shock 70 kDa protein 1A (HSPA1A) mRNA through m6A modification and inhibited osteoblast senescence.^502^ Furthermore, m6A also regulates osteoblast differentiation and bone formation through multiple signalling pathways, including BMP/Smad, PI3K/AKT and Wnt/β‐catenin.^503^

*Metformin*: This drug has shown good effects in anti‐senescent osteoblast lineages and promoting bone formation. Kuang et al.^504^ found that metformin could prevent the senescence of hPDLCs under oxidative stress and had a protective effect on their osteogenic potential. It could also repair mitochondrial damage and inhibit osteoblast apoptosis by up‐regulating SIRT3 expression through the activation of the PI3K/AKT signalling pathway.^505^ Zhou et al.^506^ found that metformin reversed oxidative stress induced by high glucose and promoted osteoblast differentiation of BMSCs through the ROS–AKT–mTOR axis in vitro.

Extracts from certain Chinese herbs or plants also have been proven to have anti‐ageing and osteogenic effects. Studies have shown that Resveratrol can activate SIRT1 through the PI3K/Akt signalling pathway, regulate autophagy in senescent osteoblasts,^338^, ^507^ regulate SIRT1 and RUNX2 to reverse TNF‐β inhibition on MSCs^508^ and improve the differentiation of senescent BMSCs into bone formation through the AMPK/ROS signalling pathway.^509^ Other examples with antioxidant, anti‐ageing and anti‐inflammatory effects include dendrobium officinale polysaccharides, pyrroloquinoline quinone, irisin, curcumin, leonurine, fisetin, luteolin and resveratrol. These extracts play roles in anti‐senescence and promoting osteogenesis in osteoblast lineages through different signalling pathways.^380^, ^418^, ^510^, ^511^ With advances in biomaterials, some biomaterials are also used in research on anti‐senescent osteoblasts. Cerium oxide nanoparticles, for instance, can significantly enhance the osteogenic mineralisation ability of MC3T3‐1 cell senescence models induced by ionising radiation and H_2_O_2_ and show a protective effect on chromosome fragmentation.^512^

## 6 CONCLUSIONS AND OUTLOOK

Faced with the global ageing, crisis, age‐related chronic diseases pose a serious threat to human health. Existing studies have shown that osteoporosis is closely related to ageing and the senescence of osteoblasts in the bone microenvironment. Counteracting osteoblast senescence and balancing the differentiation, proliferation and function of osteoclasts and osteoblasts will remain central to age‐related osteoporosis research.

During ageing, osteoblast lineages undergo significant changes that affect their ability to form, maintain and repair bone. Osteoblast precursors, including MSCs, show decreased proliferative capacity and multifunctionality, resulting in impaired osteogenic differentiation potential. The biological behaviours and functions of senescence‐related osteoblast lineages are regulated by a variety of signalling pathways associated with ageing, which may influence the cell cycle, oxidative stress response and cell metabolism. In short, the proliferation ability of senescent osteoblast lineages is weakened, affecting the renewal and repair of bone tissue. Moreover, the mineralised bone formation process is also negatively affected by ageing, resulting in abnormal bone matrix formation and mineralisation. This further leads to an imbalance in bone homeostasis in the body and ultimately accelerates bone loss.

Anti‐senescence interventions targeting osteoblasts could potentially revolutionise the treatment and prevention of osteoporosis. For instance, pharmacological agents that inhibit senescence‐associated pathways, such as mTOR inhibitors or senolytics, have shown promise in preclinical studies by enhancing osteoblast function and bone formation. Similarly, lifestyle modifications, including CR and regular physical exercise, have been demonstrated to mitigate osteoblast ageing and improve bone health. Moreover, the development of novel biomarkers for osteoblast senescence could facilitate early diagnosis and personalised treatment strategies for osteoporosis.

However, cellular senescence is a double‐edged sword. It is a natural physiological phenomenon in biological development and plays an important role in certain physiological processes. For instance, senescent cells ensure correct limb patterning during embryogenesis, and placental senescent cells release factors that promote childbirth.^513^ Blindly combating senescent cells can have detrimental effects on human health. Future research should focus on further lucidating the molecular mechanisms of senescence‐related osteoblast lineages, particularly how these signalling pathways regulate osteoblast differentiation, proliferation and mineralisation. It is necessary to identify and validate biomarkers of senescence‐related osteoblast lineages to enable early identification and assessment of osteogenic decline and accurately predict the risk of age‐related bone diseases. Developing intervention strategies for senescence‐related osteoblast lineages, including cell therapy, gene therapy and drug therapy, will aim to achieve personalised interventions to restore osteoblast function and improve bone tissue health. From exploring mechanisms and conducting animal research to advancing clinical studies, benefiting humankind is the ultimate goal of scientific research. Building on previous research results, large randomised controlled trials are needed to evaluate and ensure the safety, benefits and side effects of anti‐ageing treatments, thereby validating the reliability of early clinical trials. With the advancement of AI, the development of multi‐modal, multi‐omics and multi‐species AI models, such as Precious3GPT, undoubtedly provides valuable assistance for the research of age‐related diseases.

## AUTHOR CONTRIBUTIONS

C. Y., K. W., S. L. and G.L. conceived the study. Z. Z., P. L. and Y. S. wrote the original draft. L. M., Y. X., J. L., B. T., D. Z., H. L., H. W., X. Z., Z. O., J. W., H. X., D. W., S. P., Y. D., Z. D., B. W., Z. L., W. K., K. Z., X. X., X. F., L. T., G. L., S. L., K. W. and C. Y. reviewed and edited the manuscript. All authors have read and approved the article.

## CONFLICT OF INTEREST STATEMENT

The authors declare no conflicts of interest.

## ETHICS STATEMENT

The authors have nothing to report.

## CONSENT

The authors have nothing to report.

## Data Availability

The data that support the findings of this study are available from the corresponding author upon reasonable request.
